# Reasoning and empathy are not competing but complementary features of altruism

**DOI:** 10.1093/pnasnexus/pgag015

**Published:** 2026-01-20

**Authors:** Kyle Fiore Law, Stylianos Syropoulos, Paige Amormino, Abigail Marsh, Liane Young, Brendan Bo O’Connor

**Affiliations:** School of Sustainability, College of Global Futures, Arizona State University, Tempe, AZ 85287, USA; School of Sustainability, College of Global Futures, Arizona State University, Tempe, AZ 85287, USA; Department of Psychology, Georgetown University, Washington, DC 20007, USA; Department of Psychology, Georgetown University, Washington, DC 20007, USA; Interdisciplinary Neuroscience Program, Georgetown University, Washington, DC 20007, USA; Department of Psychology & Neuroscience, Boston College, Chestnut Hill, MA 02467, USA; The Schiller Institute for Integrated Science and Society, Boston College, Chestnut Hill, MA 02467, USA; Department of Psychology, University at Albany, State University of New York, Albany, NY 12222, USA

**Keywords:** empathy, reasoning, effective altruism, extraordinary altruism, equality

## Abstract

Humans can care about distant strangers, an adaptive advantage that enables our species to cooperate in increasingly large-scale groups. Theoretical frameworks accounting for an expansive moral circle and altruistic behavior are often framed as a dichotomy between competing pathways of emotion-driven empathy versus logic-driven reasoning. Here, in a pre-registered investigation comparing variations in empathy and reasoning capacities across different exceptionally altruistic populations—effective altruists (EAs) who aim to maximize welfare gains with their charitable contributions (*N* = 119) and extraordinary altruists (XAs) who have donated organs to strangers (*N* = 65)—alongside a third sample of demographically similar general population controls (*N* = 176), we assess how both capacities associate with altruistic behaviors that transcend conventional parochial boundaries. We find that, while EAs generally manifest heightened reasoning ability and XAs heightened empathic ability, both empathy and reasoning independently predict greater engagement in equitable and effective altruism on laboratory measures and behavioral tasks. Interaction effects suggest empathy and reasoning, when combined, often predict the strongest willingness to prioritize welfare impartially and maximize impact. These results suggest complementary candidate roles for empathy and reasoning in overcoming biases that constrain altruism, supporting a unified framework for expansive altruism and challenging the empathy-reasoning dichotomy in existing theory.

Significance Statement This research employs a unique special-population approach to begin resolving a longstanding theoretical debate on the roles of reasoning and empathy in altruism. By comparing for the first time effective altruists (EAs)—who maximize welfare through evidence-based, stranger-directed giving—and extraordinary altruists (XAs)—who have donated organs to strangers—to demographically similar controls, we illuminate distinct candidate cognitive and affective pathways toward impartial, high-impact altruism. Our findings call into question the conventional empathy-reasoning dichotomy, suggesting these capacities may function synergistically to reduce parochial biases and support more equitable, effective forms of giving. This work lays the foundation for a unified theoretical framework that explains how individuals transcend social boundaries to prioritize the welfare of distant others for the greater good.

## Introduction

Humans possess an unprecedented capacity to help, up to and including saving the lives of distant strangers, thanks to advances in technology, economics, and medicine (1–3). However, this capacity is often underutilized. Although understanding and reducing various forms of parochial bias has been a key aim of research in psychology and behavioral science over the past half-century (4–6), inequality in altruism still persists. People routinely prioritize caring for socially close individuals, such as family and friends, and are far less likely to extend help to distant, unrelated others (7–9). But overcoming inequality in care and altruism may be necessary for tackling major societal challenges, such as global poverty, hunger, preventable diseases causing early mortality, and the disproportionate impacts of climate change on marginalized populations. These challenges require both individual and collective action to help others *equitably* in order to maximize welfare and reduce suffering most *effectively* (10–14). What features of the human mind give rise to altruistic equity—impartial concern for others across social, spatial, and temporal divides—and altruistic effectiveness—a focus on maximizing welfare for the greatest number?

While empathy has traditionally been championed as a prosocial force (15, 16), more recent theoretical perspectives within psychology (10, 17, 18) and moral philosophy (12, 13) suggest that empathy—particularly affective empathy, as it is conceptualized here—hinders impartial altruism. These perspectives propose deliberative reasoning, guided by utilitarian principles, as a more reliable foundation for achieving altruistic equity. This theoretical framing, which positions empathy and reasoning as opposing forces, has gained significant traction in philosophical and psychological discussions, particularly within the context of “effective altruism”. Effective altruism, a social movement at times associated with utilitarianism, emphasizes using evidence and reason to maximize the welfare impact of altruistic actions, often by prioritizing causes that benefit socially and geographically distant individuals in the greatest need (12). Within (13) and beyond (19, 20) the effective altruism movement, scholars increasingly frame empathy as inherently biased and limited in its ability to expand care and altruism to distant others (10, 21). This growing emphasis on reasoning represents a theoretical shift away from the traditional view, which considers empathy to be the cornerstone of prosocial behavior (15, 16, 22).

However, emerging empirical research on extraordinary altruists, such as organ donors who undergo great personal costs to help strangers, challenges the shift away from empathy as a force for prosocial good. Rather than showing *reduced* empathy, these individuals exhibit *heightened* empathic responses to the suffering of psychologically distant others (23). Consistent findings of empathic enrichments within this group suggest that empathy can play a critical role in motivating impartial care (24–26). Despite this, research on organ donors has predominantly focused on emotional abilities, leaving the role of rational abilities in extraordinary altruism largely unexplored. Conversely, self-identifying adherents to the effective altruism movement remain understudied altogether, but particularly with regard to their emotional capacities, as much of the existing philosophical discourse surrounding effective altruism prioritizes reasoning over empathy.

Here, we study extraordinary altruists and effective altruists, two hyper-altruistic populations who both prioritize helping distant others, to better understand the features that might promote exceptional altruism in the general population. This approach builds on a well-established methodology: research on experts often reveals key insights into general cognitive or behavioral phenomena. For example, studies of professional taxi drivers—experts in spatial navigation—have illuminated the understanding of spatial reasoning and memory in the general population, including the neural adaptations associated with navigation expertise (27). Similarly, research on professional artists has deepened our understanding of creativity and its variability among typical adults (28). In the same way, we aim to apply this expert population approach to learn more about the cognitive and affective features that foster altruistic equity (impartial concern for others) and altruistic effectiveness (maximizing welfare) in everyday contexts by studying exceptional prosociality. By doing so, we seek to work toward reconciling theoretical ambiguity about the roles of reasoning and empathy in promoting altruism.

Although extraordinary altruists and effective altruists are not the same, they share a critical commonality: engaging in costly prosocial behavior that transcends the parochial biases often observed in the general population (18, 26). Parochial bias, frequently attributed to empathy by critics, constrains altruistic behavior to those who are psychologically closer. The existing literature, however, leaves a significant gap—it remains unclear whether the equitable and effective prosocial actions of these two populations are primarily driven by empathy, reasoning, or a combination of both. By studying both groups in the laboratory for the first time, this research aims to address these unanswered questions. Specifically, it seeks to provide novel insights into the potential roles emotional and rational pathways might play in driving exceptional altruism, while contributing to broader debates about whether empathy inherently limits prosociality (10, 21) or can instead serve to promote equity and effectiveness in altruistic behavior.

### Foundational perspectives suggest empathy is a force for prosocial good

Foundational research on the psychology of prosocial behavior supported the notion that empathy serves as a force for good with vast potential to promote altruism in general—moving concern from oneself to others—as well as across social, geographic, and ideological divides. In this vein, numerous studies have found a connection between experimentally induced empathy and subsequent acts of altruism, including altruism for strangers (15, 16, 22, 29). Research examining empathy's impact on intergroup relations suggests empathic perspective-taking and affect sharing can mitigate prejudice toward outgroup members (30–33), as well as toward distant others more broadly and historically marginalized groups (34, 35). Similarly, investigations into individual differences in empathy have yielded findings regarding its prosocial benefits (36–38), showing that individuals with greater empathic capacities tend to behave more prosocially towards others, even those who are socially distant or otherwise dissimilar.

Of course, this body of research cannot speak to the role of empathy in promoting equitable and effective altruism in the context of altruistic tradeoffs as they often manifest in the real world, where helping most effectively requires favoring helping distant others over less effective forms of helping toward those who are close (13, 17). Nonetheless, this work collectively suggests that empathy may occupy a significant place in the theoretical framework of human prosociality, enabling individuals and groups to feel more concern for others and, in turn, foster altruistic behavior across group boundaries.

### Empathic bias and the emphasis on reasoning

Despite foundational research showing that empathy can promote prosocial behavior, more recent scholarship offers a nuanced theoretical perspective, highlighting that empathic responses are often selective and constrained. This perspective is underscored by work on the Identifiable Victim Effect (39, 40), which encompasses a range of phenomena, including stronger emotional responses when beneficiaries are concretely identified (e.g. by name, photo, or personal narrative) and when the reference group is small. Similarly, research on scope insensitivity suggests that empathy does not scale proportionally with the number of people affected, leading to diminished concern for large-scale suffering (41–43). Both effects have traditionally been viewed as psychological biases that can distort utility-maximizing prosocial behavior. Furthermore, empathy is often more easily extended toward close others, like family members and friends, over distant strangers (8, 44–46) and toward those who share similarities with oneself over those who are more dissimilar (38, 47).

So, while earlier research emphasized empathy as a key driver of prosocial behavior, recent findings and theoretical frameworks suggest that empathy's biases limit its utility in promoting impartial altruism. But rather than focusing on mitigating these biases to broaden altruism's reach, many scholars in psychology (10, 17, 18, 48) and philosophy (12–14, 49, 50) have increasingly advocated for minimizing empathy's role altogether. These perspectives prioritize rational, consequential reasoning as a more effective mechanism for maximizing the preservation of lives—especially those in critical need, regardless of social proximity or relatedness (17, 20, 51–54). This theoretical shift asserts a fundamental opposition between deliberative reasoning and empathic emotion in altruistic and ethical decision-making aimed at benefiting the greater good, marking a stark contrast to earlier research that framed empathy as essential to transcending parochial boundaries and fostering altruism (55).

### A possible plurality of pathways toward altruistic equity and effectiveness?

While deliberative reasoning may be important for evaluating which among multiple causes will maximize welfare most effectively, the shift away from empathy risks undervaluing empathy's influence on altruistic decisions. Biased or not, empathy wields power over whether people allocate resources to others at all rather than keeping them—thus, donors often prioritize emotionally moving causes, even when those causes are demonstrably less effective (56). This compelling motivational potential of empathy raises critical questions about whether it can complement or enhance the effect of deliberative reasoning to foster greater altruistic equity and effectiveness or whether it is necessarily a countervailing force.

Importantly, emerging evidence challenges the idea that empathic bias is inevitable or immutable. Studies suggest that these biases often reflect broader social attitudes rather than inherent limitations of empathy itself (38, 57). Moreover, empathy can be expanded, at least temporarily, through interventions leveraging perspective-taking, narrative-building, imagination, and other practices that foster connection to distant or faceless others (58–62). These findings suggest that empathy's potential limitations can be mitigated, allowing it to contribute meaningfully to equitable prosocial outcomes and a place within contemporary theoretical discussions regarding human prosocial behavior.

Most profoundly, empirical research on extraordinary altruists provides robust evidence that empathy can drive equitable prosocial behavior across parochial boundaries, even when it comes at a great cost to oneself. Living organ donors, for example, consistently demonstrate heightened empathic responses, including increased amygdala activation and self-other neural overlap in the empathy network when witnessing others' suffering (63, 64). These findings challenge the perspective that unbiased altruism stems purely from reasoning and suggest that empathy can play a pivotal role in motivating impartial and effective helping.

Of course, the merits of reasoning warrant consideration alongside empathy, as evidence suggests both capacities may play a role. Rational and emotional appeals have been shown to motivate altruistic behavior (65), though their influence on tradeoff decisions between effective causes benefiting distant others and less effective causes benefiting close others remain understudied. And although emerging findings reveal that philanthropists exhibit not only heightened reasoning abilities but also enriched emotional capacities (66), this research has not focused on these capacities in the context of equitable and effective prosocial outcomes. These observations underscore the need to investigate how empathy and reasoning interact to foster altruistic equity and effectiveness. Could empathy and reasoning together overcome the limitations of parochial biases, driving forms of altruism that bridge social divides and maximize collective welfare? And might these capacities, when combined, reveal a synergy that reshapes our understanding of how humans achieve the most impactful and equitable prosocial outcomes? Exploring whether their relationship is interactive may reveal whether empathy and reasoning provide complementary—rather than opposing—pathways to impactful altruism.

Here, we aim to test competing perspectives on the prosocial merits of empathy and reasoning by examining their individual and interactive roles in the altruistic decisions of effective altruists (EAs), extraordinary altruists (XAs), and demographically similar controls (see Table S1 in the Supplementary Online Materials [SOM] for an in-depth description of the three samples). While recent perspectives emphasize deliberative reasoning, research on XAs suggests that empathy also plays a critical role in equitable prosociality. By studying these groups alongside controls, we aim to determine whether these pathways operate independently, in opposition, or synergistically within each population. First, we validate the exceptional altruism of EAs and XAs by measuring indicators of equitable, effective, and general prosociality. Next, we compare empathic and reasoning abilities between the groups to identify which capacities are heightened in these special populations. Finally, we examine how these abilities relate to prosociality within each group. In doing so, we seek to clarify whether empathy and reasoning complement each other in how they associate with impartial, impactful altruism, whether one pathway is more central overall, or whether distinct pathways manifest depending on the population in question. More broadly this research seeks to addresses whether framing empathy and reasoning as opposing forces may reflect a false dichotomy that impedes understanding in the prosocial domain.

## Results

### Population differences in equitable and effective prosociality

To formally investigate the architecture of prosociality among exceptional altruists and the general population, we included a battery of metrics capturing attitudes, judgments, decisions, and behaviors aligned with equitable and effective prosociality, as well as prosociality more broadly. We hypothesized that both samples of altruists would score higher on each of these metrics, which are outlined in Table S2 in the SOM. The analyses presented in the main text compare each special population to the entire control sample. An exploratory series of analyses, available in the “Supplementary Analyses” on the OSF, compares each special population to a subset of the control sample matched to each special population's demographics. The general patterns remain consistent across both series of analyses, but the findings presented here focus on the full control sample.

We began by comparing the three samples on their reported engagement in real-world charitable actions^a^, as real-world prosociality takes on numerous forms in ordinary adults (67), from financial contributions to volunteerism. Effective altruists (EAs) aim to do good primarily through financial donations to impactful causes (50, 68). By contrast, extraordinary altruists (XAs)—who earn their designation through substantial sacrifices such as donating parts of their bodies to strangers—are not necessarily unified by a commitment to financially benefiting others like EAs (23). To capture diverse forms of real-world prosociality, participants reported both the proportion of income donated and time spent volunteering annually (Real-World Charitable Action [RWCA]). One-way ANOVAs with Bonferroni corrections confirmed the exceptional altruism of EAs (*M*_Money_ = 15.57, *SD*_Money_ = 17.98; *M*_Time_ = 24.41, *SD*_Time_ = 26.33) and XAs (*M*_Money_ = 10.49, *SD*_Money_ = 9.68; *M*_Time_ = 21.66, *SD*_Time_ = 21.50), who reported donating significantly more money and volunteering more time than general population controls (*M*_Money_ = 3.68, *SD*_Money_ = 5.39; *M*_Time_ = 9.10, *SD*_Time_ = 12.89; see Table S3 in the SOM and Fig. S1). Effect sizes were large (*Cohen's d* = 0.580–1.013), and while EAs donated a higher percentage of income than XAs (15.5% vs. 10.5%), there was no significant difference in volunteering time.

These initial findings serve as an initial validity check, providing evidence that both special populations are indeed more generous overall than controls. They also suggest that the exceptional altruism of XAs is not limited to a single costly act of altruism. Rather, it runs deeper, as they report devoting substantially more of their time and money to benefit others than controls in their daily lives.

The findings above shed light on how the three samples differ on real-world prosocial behavior overall. Nonetheless, the particular focus of the present investigation is to shed light on the architecture of prosocial attitudes and actions that are *equitable*, in that they are not constrained by parochial biases that routinely limit everyday prosocial displays toward close, identifiable or otherwise similar beneficiaries (9, 10, 17, 18, 36, 45, 69), and *effective*, in that they have a high potential for maximizing welfare gains. To mimic the types of tradeoffs between social closeness and gains in welfare that are common in the real world, where resources—from the perspective of the affluent—can do greater good when donated to distant strangers, a subset of the prosociality metrics captured attitudes toward a modality of altruism that is simultaneously equitable and effective. Moreover, while the measures discussed above rely on self-reports, the metrics that follow incorporate behavioral tasks and laboratory measures to provide a more objective assessment of prosociality.

On each of these behavioral tasks, there was a significant omnibus effect of sample, with 10.0 to 18.5% of the variance in these outcomes being attributable to differences between the three subject groups (see Table S3 and Fig. S2 in the SOM). On the Moral Judgment Vignettes (MJV) task, both EAs and XAs reported more positive third-party moral judgments of welfare-maximizing altruism (i.e. effectiveness) directed toward distant strangers that came at the expense of not being able to help a less-effective but socially closer alternative (i.e. equitability). On balance, subjects from the two special populations did not differ from one another in their judgments of these altruistic decisions. Similarly, both special populations made a greater number of decisions in the Social Discounting Task (SDT) to allocate larger monetary rewards (i.e. effectiveness) to other individuals across a range of social distances (i.e. equitability) rather than keeping smaller rewards for themselves. Especially intriguing is that XAs demonstrated greater prosociality than EAs on this task. Overall, these findings provide support that the moral judgments and prosocial decisions of both special populations favor equitability and effectiveness in altruism in the context of real-world tradeoffs—where supporting the most effective causes also happens to require a departure from the parochial biases which generally constrain prosociality toward those who are close.

We also included measures that capture equitable and effective prosociality in isolation, to disentangle whether and how the three samples differ on each facet when considered separately. On the expansive altruism subscale of the Effective Altruism Interest Scale (EAIS), which captures impartiality regarding the relational proximity of prosocial beneficiaries—a measure that uniquely captures altruistic equity, both special populations scored higher on average than general population controls, with EAs scoring higher on average than XAs (see Table S3 and Fig. S2 in the SOM).

On the effectiveness focus subscale of the EAIS, however, EAs scored higher than both general population controls *and* XAs, with XAs scoring even lower than members of the general population. This finding deviates from our pre-registered hypothesis and the earlier results on measures capturing equitability and effectiveness together. However, it aligns with broader distinctions between the two groups of altruists. EAs explicitly prioritize consequentialist principles, emphasizing impact and effectiveness as codified rules, whereas XAs show a stronger alignment with equitability in their real-world prosocial actions (i.e. donating organs to strangers), with less explicit focus on maximizing effectiveness. Yet, on a behavioral metric of effectiveness prioritization—the Behavioral Donation Task or BDT, where participants allocated resources between effective and ineffective causes across 16 trials—both EAs and XAs chose the effective charitable option more frequently than the control sample (see Table S3 and Fig. S2 in the SOM). These findings suggest that extraordinary altruists lean toward effective causes in their behaviors, decisions, and judgments, even if this is not consistently reflected in their explicit attitudes.

In summary, the findings largely support our pre-registered predictions. Both EAs and XAs scored higher than members of the general population on six out of seven prosociality measures, indicating greater equitable and effective prosociality, as well as broader prosocial tendencies. While EAs outperformed XAs in measures focusing on effectiveness, this pattern did not hold on the BDT, a behavioral metric of effective prosociality. Together, the findings highlight that both effective and extraordinary altruists demonstrate prosociality in their real-world behaviors and in laboratory settings. Although XAs' real-world altruism—organ donation to strangers—aligns more closely with altruistic equity in overcoming parochial bias, their behavior also reflects a preference for impactful causes.

### Population differences in empathic and reasoning ability

After finding evidence that both EAs and XAs exhibit equitable and effective prosociality in the lab, we examined differences in empathic and reasoning abilities, key features in debates on prosociality across psychology and philosophy. Namely, prosocial behavior is often shaped by parochial tendencies, favoring those who are relationally close or similar (5, 30, 38, 70), even when helping distant others could achieve greater good (9). Empathy is a key driver of prosocial actions (15, 55, 71). However, like prosociality more broadly, empathy is often expressed in a parochial pattern—it is more easily felt for those who are closer and more similar (19, 38). Psychological inquiry has long sought to address these biases to promote greater equity in altruism (5, 30, 55). Yet, some researchers and philosophers, including proponents of effective altruism, have argued that empathy itself is to blame for parochial bias, encouraging would-be donors to downregulate empathic emotion in favor of rational, deliberative decision-making as a way to mitigate parochial tendencies and expand the scope of altruism (10, 18, 21, 51).

Despite prevailing skepticism regarding empathy's potential to promote altruism at a distance, emerging evidence suggests that empathy can be a force for equitable and effective good (36, 59). Of particular relevance, empathy has been shown to drive prosocial attitudes and behaviors even toward distant strangers among altruistic organ donors (i.e. extraordinary altruists or XAs (69)). Moreover, this exceptionally altruistic population has been shown to possess profound enhancements in empathic ability compared to general population controls. While proponents of effective altruism advocate reasoning over empathy as a better guide for equitable and effective altruism, the research on XAs raises the question of whether effective altruists (EAs)—who have been empirically understudied to date—also show similar enhancements in empathic ability, and whether empathy drives their prosocial engagement. It is also possible that reasoning ability is heightened among EAs exclusively, or among EAs and XAs collectively, and represents a distinct or complementary route, alongside empathy, in fostering altruistic equity and effectiveness.

Given conflicting theory and evidence in the extant literature, we wagered three alternative hypotheses: Relative to general population controls, (i) consistent with EA discourse and research on XAs, EAs might score higher on reasoning ability and XAs higher on empathic ability; (ii) considering the abundance of evidence for heightened empathy among XAs and limited evidence on the cognitive and affective profiles of EAs, both altruistic populations might score highly on measures of empathic but not reasoning ability; or (iii) EAs and XAs might score higher on measures of empathic and reasoning ability alike.

**Fig. 1.**
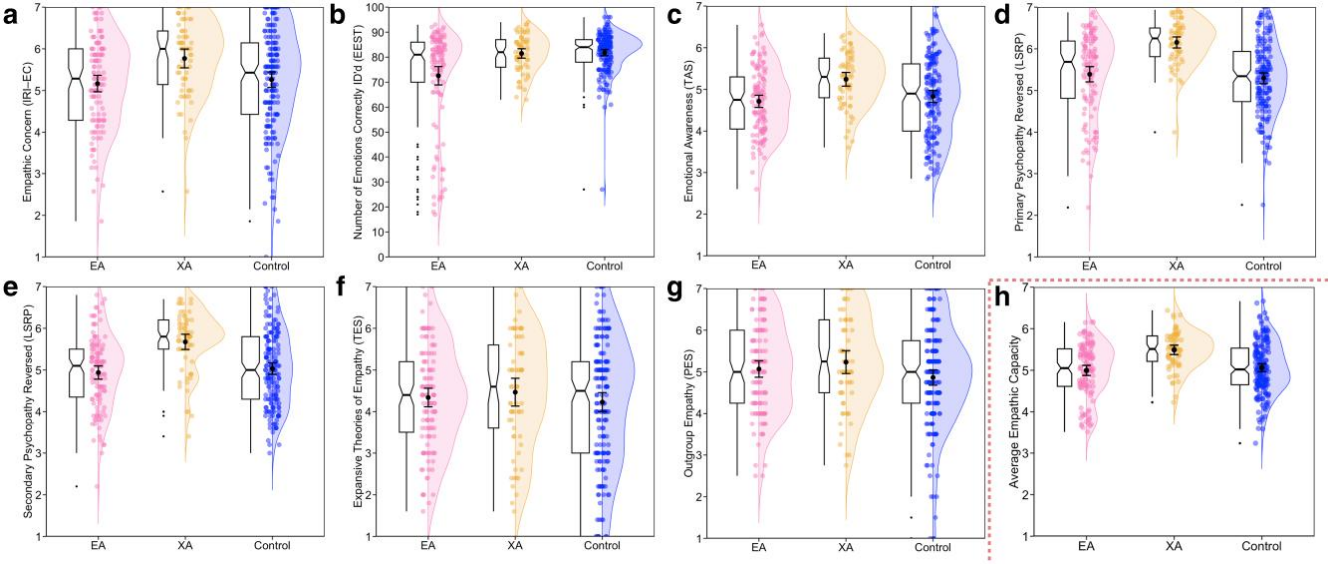
Differences between effective altruists, extraordinary altruists and general population controls on measures capturing empathic ability. Raincloud plots displaying empathic concern on the IRI (a), the number of correctly identified emotions on the EEST (b), alexithymia reverse coded to convey emotional awareness on the TAS (c), primary and secondary psychopathy reverse coded to convey emotional ability on the LSRP (d and e), beliefs that empathy is malleable on the TES (f), outgroup empathy on the PES (g), and, for the sake of visualizing the overall pattern across measures, the average across all measures of empathic ability (h; EEST was transformed to be on a 1–7 scale prior being averaged). Plots display individual data-points, jittered for readability, with overlaid split-violins to illustrate the shape of the underlying probability distributions. Means and error bars depicting 95% CIs are also included, as well as box plots with notches to convey 95% CIs around the medians.

Our results showed that with regard to empathic ability, members of the three populations differed on several measures: empathic concern (EC) on the Interpersonal Reactivity Index (IRI), capacities in correctly identifying emotional content in written statements on the Emotionally Evocative Statements Task (EEST), alexithymia on the Toronto Alexithymia Scale (TAS), and primary and secondary psychopathy on the Levenson Self-Report Psychopathy (LSRP) scale. For the latter three measures, higher scores (reverse-coded) correspond to greater empathic ability. The three populations did not differ significantly on beliefs regarding the within-person malleability of empathy on the Theories of Empathy Scale (TES), nor on feelings of outgroup empathy on the Parochial Empathy Scale (PES; though results trended toward significance on the PES, with XAs scoring marginally higher than controls^b^). Confirming the findings from research on altruistic organ donors (23, 69), XAs, relative to controls, scored higher on EC on the IRI, reverse-coded alexithymia on the TAS, and reverse coded primary and secondary psychopathy on the LSRP. Moreover, on each of these metrics, as well as the EEST, XAs scored higher than EAs, though there was no significant difference in performance on the EEST between XAs and controls. Intriguingly, EAs, relative to controls, showed deficits in emotion recognition on the EEST. And, while EAs differed significantly from controls on no other metric, they trended lower on most (see Table S4 in the SOM and Fig. 1).

**Fig. 2.**
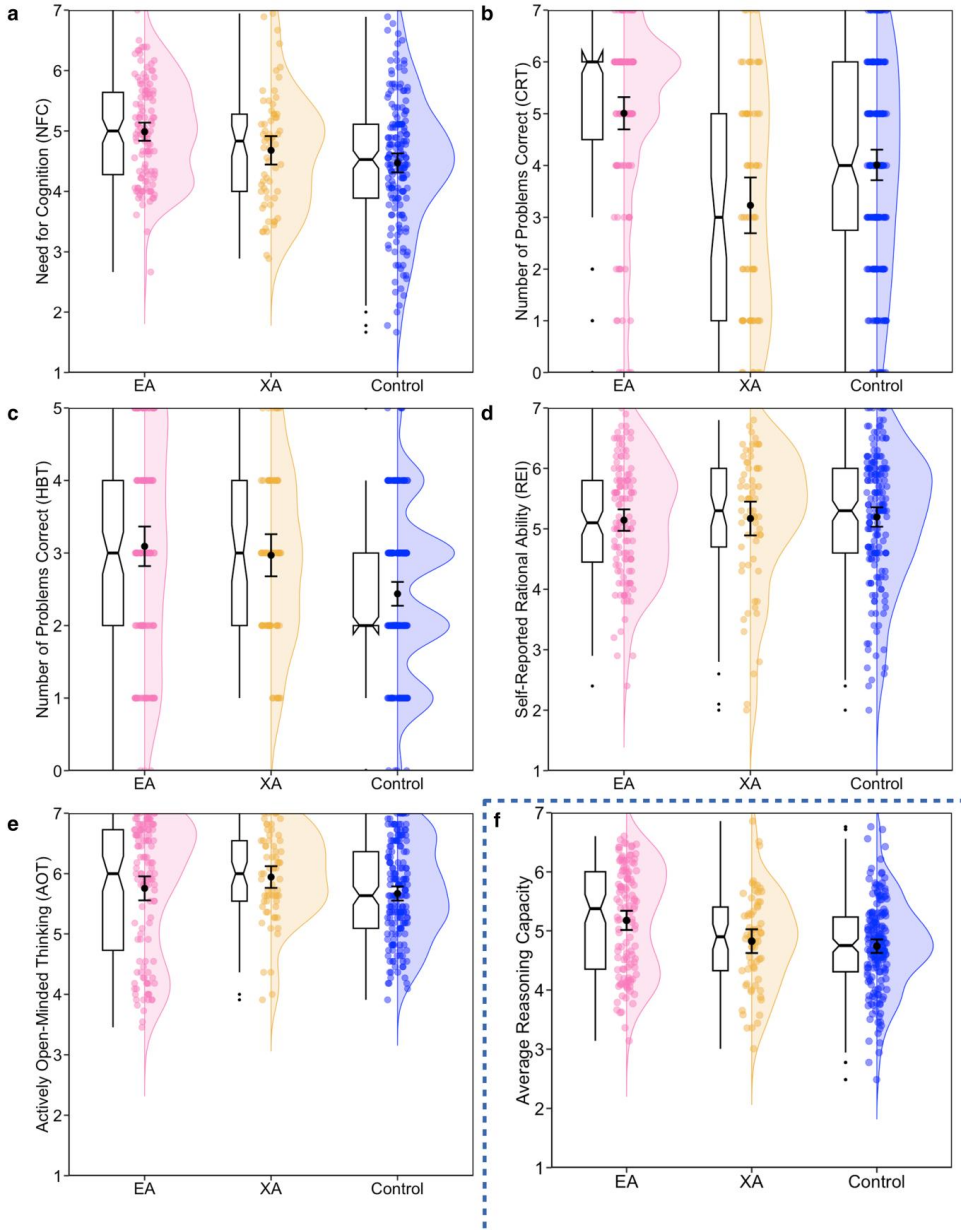
Differences between effective altruists, extraordinary altruists, and general population controls on measures capturing reasoning ability. Raincloud plots displaying need for cognition on the NFC (a), the number of correctly solved problems on the CRT (b) and HBT (c), self-reported rational ability on the REI (d), actively open-minded thinking (AOT; e), and, for the sake of visualizing the overall pattern across measures, the average across all measures of reasoning ability (f; CRT and HBT were transformed to be on 1–7 scales prior being averaged). Plots display individual data-points, jittered for readability, with overlaid split-violins to illustrate the shape of the underlying probability distributions. Means and error bars depicting 95% CIs are also included, as well as box plots with notches to convey 95% CIs around the medians.

With respect to reasoning ability, the three populations differed meaningfully on Need for Cognition (NFC) and tendencies to engage in deliberative processes to arrive at correct answers to challenging word problems on both the Cognitive Reflection Test (CRT) and a battery of Heuristics and Bias Tasks (HBT). No significant differences were observed among the populations in self-reported reasoning ability on the Rational Experiential Index (REI), nor in beliefs about changing one's mind to accommodate evidence that challenges existing viewpoints on the Actively Open-Minded Thinking (AOT) scale. Intriguingly, and largely consistent with perspectives advanced among philosophers and researchers associated with the effective altruism movement, EAs scored higher than controls on NFC, the CRT, and the HBT. Moreover, XAs scored lower than EAs and controls on the CRT, but higher than controls on the HBT. This suggests that while EAs exhibit a strong tendency towards deliberative reasoning across various measures, XAs may engage in greater reasoning relative to controls in specific contexts, highlighting distinct cognitive profiles between these groups (see Table S4 in the SOM and Fig. 2).

In summary, these findings align with previous research on altruistic organ donors (XAs), highlighting their heightened empathic abilities (23). However, they provide new insights into their cognitive profiles, which remain understudied. XAs report similar enjoyment of effortful thinking (NFC) as the general population but perform worse on the Cognitive Reflection Test (CRT), which measures the ability to override intuitive but incorrect answers. Interestingly, they outperform the general population on heuristics and bias tasks, which are more challenging but less reliant on suppressing intuitive responses relative to the CRT (72–75). Thus, the key difference between EAs and XAs may lie in their *motivation* to engage in effortful thinking rather than reasoning *ability* itself. While both groups show unique cognitive strengths, their *preferences* for challenging cognitive tasks differ significantly.

Furthermore, these findings offer new insights into the cognitive and affective profiles of EAs, revealing their heightened reasoning ability compared to controls and reduced empathic ability relative to XAs. In fact, EAs sometimes score lower than the general population in specific aspects of empathy, such as emotion identification. So, while both groups excel in equitable and effective altruism, they manifest divergent trait levels of cognitive and affective abilities: EAs favor reasoning, while XAs excel in empathy. And, because both altruistic populations manifest higher levels of altruistic equity *and* effectiveness on the behavioral tasks measured here, these findings suggest that reasoning and empathy may not be opposing forces but perhaps alternative pathways to altruistic equity and effectiveness.

### The cognitive and affective architecture of equitable and effective altruism

**Fig. 3.**
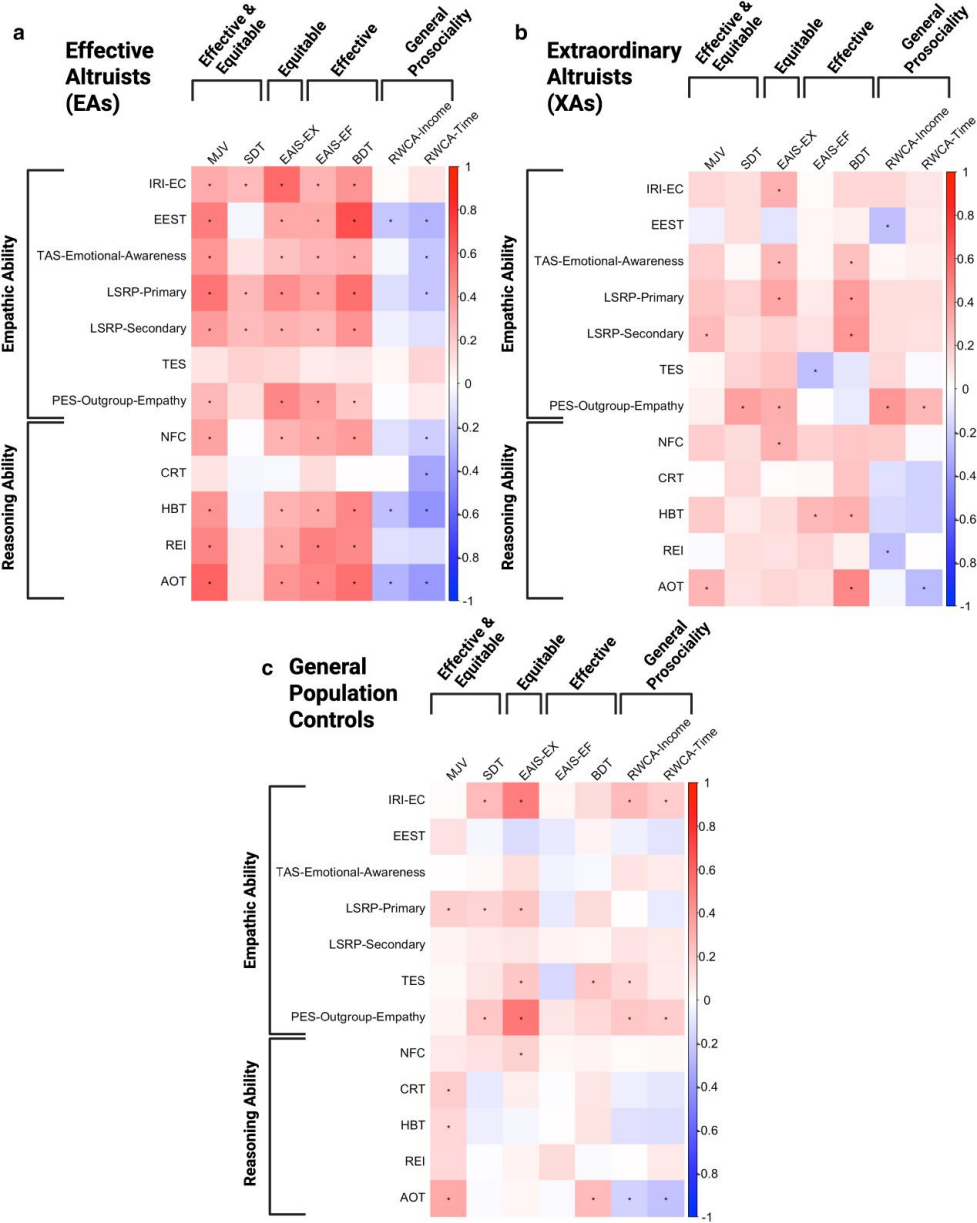
Bivariate relationships between empathic ability and reasoning ability with equitable, effective and overall prosociality among EAs, XAs, and controls. Heatmaps displaying correlation coefficients from −1 (blue) to +1 (red) in EAs (a), XAs (b), and controls (c). Asterisks correspond to statistically significant relationships. Although secondary to the primary focus of the investigation, we included the “Personal Distress” subscale of the IRI, pre-registering that we would evaluate associations with prosociality metrics.

To examine further whether reasoning and empathy both present candidate pathways to equitable and effective altruism, we began by estimating bivariate correlations between each measure of empathic and reasoning ability with each prosocial outcome within each sample (see Fig. 3 below and Tables S5–S7 in the SOM). In line with our predictions, across samples, most measures of empathic and reasoning ability were associated positively with most attitudes and behaviors in line with equitable and effective prosociality, though many of these relationships, particularly in the smaller sample of XAs, were nonsignificant. Intriguingly, these bivariate relationships were strongest among effective altruists, even with respect to empathic predictors. However, associations with general real-world prosociality measures (which relied on self-report and did not explicitly assess equitability or effectiveness) were often weaker or even negative. This pattern suggests that affective and cognitive abilities may be more strongly linked to the psychological architecture of equitable and effective altruism than to broadly defined prosociality. Nonetheless, it is also possible that the behavioral and task-based measures employed here provide a more valid indicator of meaningful altruistic engagement than these self-report metrics.

It is also worth noting that in the general population, empathy appeared to be a stronger predictor of equitable and effective altruism than reasoning. These findings stand in contrast to perspectives on the inherent parochiality of empathy (10) and the primacy of reasoning (18) in the prosocial context. They suggest instead that our ability to empathize with others may live among the most critical tools at our disposal—rather than among our greatest limitations—for promoting the greater good through beneficent action.

For the focal analysis, we conducted a series of seven multiple regression models per sample (one per prosocial outcome, 21 in total), entering a composite factor for empathy and a composite factor for reasoning as simultaneous predictors. The goal of estimating these models was to ascertain whether empathy, reasoning, or both accounted for unique variance in each outcome, above and beyond the effect of the alternative capacity. As pre-registered, because of the vast number of number of metrics we included to capture empathic and reasoning ability, we reduced the dimensionality of both categories of predictors (see Tables S8 and S9 and Figs. S3 and S4 in the SOM). First, we conducted exploratory factor analysis (EFA) using the maximum likelihood extraction method in combination with oblimin rotation on the battery of measures of empathic ability and reasoning ability, separately. For empathic ability, a single factor emerged, but beliefs in the malleability of empathy on the TES failed to load onto the factor, while outgroup empathy on the PES and scores on the EEST had weak loadings, below 0.5, and thus were excluded from the next stage of analysis. Likewise, for reasoning ability, each measure loaded onto a single factor, but scores on the CRT had loadings below 0.5, and thus were excluded from the next stage of analysis.

For empathic ability, scores on the TAS (reverse-coded), IRI-EC, LSRP-P (reverse-coded), and LSRP-S (reverse-coded) were retained, z-transformed, and averaged (*Cronbach's* α = 0.79). For reasoning ability, scores on the NFC, IRI-EC, AOT, and HBT were retained, z-transformed, and averaged (*Cronbach's* α = 0.69). We also assessed reliability without excluding the TES, PES, and EEST for empathic ability (*Cronbach's* α = 0.20) and without excluding the CRT for reasoning ability (*Cronbach's* α = 0.64). Because reliability was greater when including only the predictors indicated by the results of the EFA, we went forward with estimating the regression models using the composites derived from EFA (rather than separate composites including all of the predictors). Nonetheless, regression models that include all measures of empathy and reasoning as simultaneous predictors of each prosocial outcome are presented in the SOM in Table S9.

Within each sample, the multiple regression results largely confirmed that both empathic and reasoning ability may be forces for good (see Table S10 in the SOM for the results pertaining to the analyses described below, which include as predictors the empathic and reasoning ability composites derived from factor analysis and Table S9 in the SOM for results pertaining to the analyses conducted with all measures of empathy and reasoning as simultaneous predictors). Notably, scores on the empathic ability composite factor were positively and significantly associated with: (i) moral judgments of equitable and effective prosociality on the Moral Judgment Vignettes task among EAs, (ii) generosity on the Social Discounting Task among EAs and controls; (iii) attitudes aligned with expansive altruism (altruistic equity) on the EAIS among all three samples; (iv) behavioral donations to effective charitable causes among EAs and XAs; and (v) real world monetary charitable contributions (as the percentage of income donated in a given year) among controls. Beyond the findings presented above, which demonstrate that exceptionally caring individuals show enhancements in empathy, these findings suggest that among exceptional altruists and ordinary adults alike, greater empathy and emotionality often predicts greater attitudes, judgments, decisions and behaviors in line with altruistic equity, effectiveness, and real-world charitable action, above and beyond differences in reasoning ability. Critically, they provide clear evidence that arguments against empathy may be misguided (10, 13), as greater ability to empathize with others appears to associate positively with rather than inhibit boundary transcendent altruism and prosociality that maximizes welfare.

Furthermore, these results partially align with assertions raised in discourse related to the effective altruism movement (12) and with some earlier empirical findings (17) suggesting that reasoning ability underlies the prioritization of altruistic equity and effectiveness. Namely, variation on the reasoning ability composite factor was significantly and positively associated with: (i) moral judgments of equitable and effective prosociality on the MJV task among EAs and controls; the prioritization of (ii) expansiveness (equity; among EAs) and (iii) effectiveness (among EAs and XAs) in altruism on the EAIS; and (iv) behavioral donations to effective causes among EAs and XAs on the BDT. However, it is noteworthy that the associations between reasoning ability with equitable and effective prosociality were most pronounced among members of the EA subject group, who explicitly emphasize applying reasoning skills to guide altruistic decision-making.

Collectively, these findings suggest that both empathy and reasoning, rather than one over the other, are generally associated with greater altruistic equity, effectiveness, and real-world charitable action. Importantly, empathy does not consistently constrain the scope of equity and impact. However, there are exceptions, especially when examining associations with individual facets of affective ability in finer-grained detail (see Table S9 in the SOM). For instance, the tendency to accurately identify emotions in written statements (measured by the EEST) was negatively associated with generosity on the SDT among EAs and the prioritization of expansiveness in altruism among controls. These unexpected results warrant further investigation to clarify their implications.

It is also notable that controls who reported more malleable lay theories of empathy scored significantly lower in their prioritization of effectiveness in altruism. Prior research suggests that individuals with more malleable versus fixed theories of empathy exert greater empathic effort toward distant and stigmatized targets (58). Thus, this result was unexpected. One possibility is that it reflects an “empathic bystander effect” (76), wherein holding stronger beliefs about the malleability of empathy allows individuals to diffuse personal responsibility for addressing large-scale suffering. Because the measure captures beliefs about the malleability of empathy not only for oneself but also for others, individuals who score higher might overestimate others' empathic capacities, assuming others will prioritize effectiveness in their stead. Further research is needed to explore this possibility.

Deviations from expected results were observed not only in measures of empathic ability but also in reasoning ability. For instance, Actively Open-Minded Thinking (AOT)—the willingness to change one's mind when faced with new evidence—was moderately negatively associated with real-world charitable donations and volunteerism for EAs and weakly negatively associated for controls. Similar results were observed for the reasoning ability composite factor (see Table S10 in the SOM). These findings suggest that, in some contexts, reasoning abilities may backfire by increasing focus on the opportunity costs of giving. This effect may occur even among individuals who typically prioritize effectiveness in their altruistic efforts. However, these findings were exceptions to the general trend across samples and measures, where both reasoning ability and empathic ability were positively associated with equitable and effective prosocial attitudes and behaviors.

### The interactive effects of reasoning and empathy on equitable and effective altruism

For exploratory purposes, to investigate the cognitive and affective architecture of altruism in greater depth, we next investigated whether reasoning and empathy interact within each population concerning their associations with measures of prosociality. This analysis aimed to determine if the combined influence of reasoning and empathy might relate to prosocial behaviors differently than either trait alone. Twenty-one multiple regression analysis mirroring those above were estimated, this time specifying as predictors the empathic ability composite, reasoning ability composite, and their interaction. See Table S11 in the SOM for the results from these models.

Here, largely in line with the findings above (where the empathy-reasoning interaction was not accounted for), scores on the empathic ability factor were associated with greater: (i) moral acceptability judgments of equitable and effective altruism on the MJV task among EAs; (ii) generosity on the SDT among controls; (iii) attitudes aligned with expansive altruism (altruistic equity) on the EAIS among all three subject groups; (iv) behavioral donations to effective charitable causes among EAs and XAs; and (v) real-world monetary charitable contributions among controls. Likewise, scores on the reasoning ability factor were associated with greater: (i) generosity on the SDT among controls; (ii) prioritization of expansive altruism and effectiveness focus on the EAIS among EAs; and (iii) behavioral donations to effective causes among EAs and XAs. Also mirroring the findings in which the interaction was not considered, in a few instances, the reasoning ability factor showed negative associations with prosociality. Namely, reasoning ability was associated negatively with real-world monetary contributions to charity among EAs and Controls and with real-world volunteerism among EAs. Also notable is that the empathic ability factor in no instances associated significantly and negatively with any facet of prosociality in any sample.

Significant interactions were observed between empathic and reasoning abilities in a few key contexts (see Fig. S5 in the SOM). These interactions emerged in their associations with behavioral donations (BDT) and real-world monetary charitable contributions among EAs, moral judgments on the MJV task among XAs, and the prioritization of expansive and effective altruism among controls. For the BDT and MJV judgments, greater empathic ability acted as a protective factor against lower reasoning ability, such that empathy was more strongly and positively associated with prosociality at lower reasoning levels. Conversely, in the context of real-world charitable action (among EAs) and the prioritization of expansiveness (equity) in altruism (among controls), empathic ability was more strongly and positively associated with prosociality at higher levels of reasoning ability. These findings suggest that the interplay between empathy and reasoning varies across contexts and populations, with each capacity compensating for the alternative in promoting prosocial outcomes in some contexts and potentially amplifying the prosocial benefits of the alternative capacity in others.

Perhaps most intriguing was that a significant crossover interaction characterized the relationship between reasoning and empathy with the prioritization of effectiveness in altruism among the control sample. At lower levels of empathy, greater reasoning ability was linked to less prioritization of effectiveness, whereas at higher levels of empathy, greater reasoning ability predicted more prioritization of effectiveness. Similarly, empathy was negatively associated with effectiveness prioritization at lower reasoning levels but positively associated at higher reasoning levels. These findings collectively highlight the possibility that reasoning and empathy may enhance equity and effective altruism best when working together. In the general population in particular—those who comprise the majority and thus hold the greatest prosocial potential—reasoning associates with greater impact prioritization only when empathy is high, and empathy associates positively with prioritizing impact only when reasoning is also high. Although further experimental work building on the present findings is warranted, the current data underscore the value of cultivating both reasoning and empathy as complementary capacities that, together, might better promote altruistic behavior than either can alone.

## Discussion

Addressing many of the world's greatest challenges—from poverty and hunger to climate change—requires directing prosociality toward outgroups, socially distant others and statistical collectives in the greatest amount of need (77). As such, social psychologists have long sought to understand and address the biases that drive prosocial favoritism toward ingroup members, socially close others, and singular identifiable targets (5, 38). While foundational research underscores empathy's role in fostering prosocial behavior by enabling individuals to share the emotions of others in need (15, 16, 22, 29), empathy, like prosociality, is more easily elicited by relationally close or similar individuals and singular victims (8, 38, 44–47). In response to these biases, some contemporary perspectives in psychology and beyond (e.g. the effective altruism movement) advocate for combatting empathy with deliberative reasoning and evidence-based strategies to promote greater altruistic equity and effectiveness (17, 20, 51–54).

The present research employs an exceptional population approach, examining effective altruists (EAs), extraordinary altruists (XAs—living, nondirected organ donors), and general population controls to challenge the notion that empathy and reasoning are inherently at odds. Our findings reveal that both EAs and XAs, who prioritize equity and impact in their prosocial decisions and behaviors, possess enriched but distinct psychological capacities: EAs demonstrate heightened reasoning abilities, while XAs exhibit heightened empathy. However, rather than opposing one another, empathy and reasoning associate positively with altruistic equity and effectiveness across all groups and for most outcomes. By and large, these results suggest that empathy and reasoning may represent complementary forces that work together to increase equity and impact in altruistic behavior, rather than operating as competing pathways. Notwithstanding the value of further experimental research to shed light on causal directionality, these findings highlight the possibility that a theoretical reconceptualization of empathy and reasoning as synergistic contributors to altruism, rather than as opposing forces, may be in order.

The findings align with and build upon previous research and discourse on altruism—particularly studies examining the psychological underpinnings of extraordinary altruism and the philosophical tenets of effective altruism—and contribute to broader debates over the prosocial merits (or detriments) of empathy. Prior work on XAs has consistently highlighted empathy as a central driver of their prosocial tendencies, enabling them to emotionally connect with the suffering of distant and unrelated others (24, 25, 64, 69, 78). However, the role of reasoning among XAs has been understudied to date, leaving a gap in understanding how cognitive capacities might also contribute to their altruistic behaviors. Conversely, while philosophical discussions on EA (12, 50)—as well as investigations into the drivers of effectiveness prioritization among the general population (17, 18, 51, 70)—have emphasized reason and evidence as critical for driving their high-impact altruistic actions (12, 50, 68), empirical research on the EA population remains sparse, limiting our ability to validate these theoretical claims through data-driven analysis. In other words, despite the shared commitment to altruism that transcends conventional parochial boundaries among EAs and XAs, the literatures on these two exceptionally altruistic groups remain disparate and often conflicting.

By incorporating both EAs and XAs alongside general population controls, the present research helps to address these gaps, offering an empirical examination of reasoning in XAs and providing much-needed data on the psychological profiles of EAs for the first time. Indeed, we find that EAs largely perform better than controls on tasks and measures capturing reasoning ability, and we replicate prior findings of heightened empathic and affective ability among XAs. Despite differences in baseline levels of these capacities across subject groups, empathic and reasoning abilities function not as opposing or independent pathways but as complementary forces that collectively associate positively with altruistic equity and effectiveness. In some instances, one capacity appears to offset limitations in the other (e.g. prioritizing effective charitable causes in the MJV task and BDT among exceptionally altruistic groups), while in other contexts, higher levels of both capacities are associated with the strongest prosocial outcomes (e.g. the real-world prosociality of EAs and the equitable, effective altruistic attitudes of typical adults). These findings not only begin to expand our understanding of the unique psychological capacities underlying altruistic behavior among the special populations but also challenge the dichotomy (10, 19, 21) between emotion and cognition in prosocial decision-making more broadly, highlighting the potential prosocial utility of both capacities across diverse altruistic contexts.

Our interpretation of the present findings is not inherently in conflict with the extensive evidence that empathy is often influenced by parochial biases (19, 45). Rather, we argue that parochial biases are neither intrinsic to nor a necessary outcome of empathy itself. Empathy, we contend, does not drive parochial bias; instead, it likely motivates altruism—perhaps at times more powerfully than reasoning. For example, individuals often prioritize emotionally compelling causes even when evidence indicates that other causes may have greater impact (56). While our attention, beliefs and motivations can narrow the focus of empathy (38), this does not imply that empathy should always be downregulated when the goal of altruism is to maximize welfare for distant others in greatest need. Instead, it highlights the potential importance of cultivating empathy—enhancing the capacity to empathize deeply with distant others and with causes that have greater potential for impact. By broadening and strengthening empathy, we may harness its motivational power to promote both altruistic equity and effectiveness. The present findings provide initial support for this possibility, not only for those who are exceptionally altruistic but also for the broader population with untapped potential for prosocial impact.

This research also suggests that descriptive theories of prosociality may require a reconceptualization to reconcile longstanding debates about the roles of empathy and reasoning in altruistic behavior. This revised framework would view reasoning and empathy not as isolated nor competing forces but as interdependent and complementary contributors to equity and effectiveness in altruism. However, further research is critical to develop, refine, and validate such a model. Experimental work, in particular, is necessary to disentangle how empathy and reasoning operate during prosocial decision-making, rather than measuring them solely as individual differences in baseline capacities, as we did here. Future studies could investigate the situational and contextual factors that activate or suppress the dynamics between empathy and reasoning, exploring how these processes jointly shape decisions about whether to help, whom to help, how much to help, and in what ways. For example, experiments could manipulate the salience of emotional appeals or rational cost–benefit considerations to determine how these elements interact to drive prosocial outcomes in real-time decision-making.

While some work comparing the relative efficacy of rational and emotional appeals to prosocial action has already begun (65), it has yet to adequately address the context of real-world tradeoffs, such as withholding aid from psychologically close beneficiaries to prioritize the greater gains in overall welfare afforded by helping those who are distant. Future experimental research could focus on directly manipulating empathic emotion and prosocial reasoning in the context of such nuanced tradeoffs by presenting participants with scenarios that require choosing between helping individuals with whom they share a closer relational bond and allocating resources to those in greater need but at a greater psychological or social distance. Such studies would provide valuable insights into how empathy and reasoning dynamically interact under the tradeoff conditions explicitly discussed in the context of effective altruism (17, 50), where helping others most effectively requires prosociality towards those who are distant.

Future research building towards a prosociality model that unifies reasoning and empathy under the same theoretical framework should also prioritize replication of these findings across diverse populations to ensure generalizability, particularly among large samples of nonexceptional altruists in the general population. Furthermore, this model must integrate the role of parochial bias, exploring not only how it constrains the prosocial reach of empathy (8, 19, 45) but also how it influences the reasoning process. Research on self-serving and close-other serving biases highlights how parochial biases shape reasoning and decision-making, often reinforcing preferential treatment toward those perceived as similar or connected to oneself (79–84). Examples such as nationalism, confirmation bias, and the ultimate attribution error illustrate how parochiality shapes cognition. These insights, often overlooked in critiques of empathy, deserve greater attention to clarify the interplay between affect, cognition, and altruism. By examining the susceptibility of both empathy and reasoning to parochial tendencies, researchers can better identify strategies for mitigating bias and enhancing the alignment of both processes with altruistic impact. Ultimately, this program of research would provide a more nuanced and actionable understanding of how empathy and reasoning jointly contribute to prosocial decision-making, informing interventions that harness their combined potential to foster more impactful and inclusive altruism.

While maximizing altruistic impact and promoting equality in altruism may seem commendable in the abstract, the ethical foundation of effective altruism has been widely debated by scholars and the general public (85 , 86). Critics argue that EA prioritizes consequentialist decisions benefiting the greater good at the expense of personal obligations to family and friends. At its most extreme, critics contend that EA can justify harmful behaviors based on speculative utilitarian welfare gains. In response, proponents claim that humans can transcend parochial evolutionary programming that favored cooperating in small groups and emphasize that EA does not explicitly endorse instrumental harm (50, 87). However, many people morally disapprove of the form of altruism promoted by EA and impose social penalties on those who adopt it (9, 88, 89). Existing research suggests that XAs, despite engaging in costly altruism toward strangers, maintain rich and fulfilling personal relationships with close others, challenging assumptions that highly impartial altruism necessarily undermines personal bonds (90). Nonetheless, future research is essential to determine whether real-world EAs experience social consequences, such as strained personal relationships, and to examine the extent to which EAs themselves endorse instrumental harm. Such investigations could bridge descriptive and normative ethical theories of prosociality, providing a foundation for more ethically sensitive interventions and guiding how altruistic practices can be promoted without alienating individuals or undermining moral intuitions.

The broader patterns in the data confirm that EAs and XAs both endorse equity and effectiveness, with EAs showing greater reasoning ability and XAs displaying greater empathy, and that both capacities contribute to altruism. However, some findings challenge these general trends. For example, while XAs prioritize altruistic effectiveness in their decisions, judgments, and consequential behaviors, this emphasis is not consistently reflected in their explicit attitudes. Similarly, despite their heightened empathic abilities, XAs outperformed the general population on heuristics and bias tasks, a measure typically associated with reasoning ability. Additionally, certain cognitive and affective capacities were unexpectedly associated with reduced prosociality in specific contexts—for instance, beliefs in the malleability of empathy were negatively linked to explicit attitudes supporting altruistic equity and effectiveness on the EAIS, and self-reported reasoning ability negatively correlated with general prosociality. Future research could examine whether XAs prioritize equity and effectiveness intuitively, or at a more implicit level, which could explain the discrepancy between their attitudes and behaviors, or if they excel in reasoning *ability* without consistently *activating it* in decision-making contexts, as suggested by their performance on the heuristics and bias tasks. Similarly, studies could investigate whether beliefs in the malleability of empathy diffuse responsibility for prosociality or whether reasoning primarily guides where resources are donated rather than determining how much is given.

Finally, the research boasts numerous methodological strengths, such as the first-ever sample comprising EAs and XAs alike—two rare and exceptionally altruistic populations—as well as comprehensive open-science practices. However, several limitations warrant attention. First, the sample size for XA sample of living organ donors was constrained by the rarity of this population, limiting statistical power. Efforts to expand this sample in future research, through partnerships with hospitals or targeted outreach, could enhance the robustness of these findings. Additionally, while the study focused on EAs and organ donors, other altruistic populations, such as humanitarian aid workers and individuals tackling global challenges, were not included. Examining these groups could reveal additional insights into the diverse architectures of prosociality. Finally, while this study included a diverse sample spanning multiple countries, future work could benefit from larger, internationally representative samples to explore cultural differences in prosociality and their implications for global welfare initiatives.

## Conclusion

Psychologists and philosophers have often framed reasoning and empathy as being in opposition when it comes to promoting equality and encouraging effective altruism. However, the findings here suggest this divide may be misplaced. While effective altruists (EAs) lean on reasoning and extraordinary altruists (XAs) demonstrate the power of empathy, neither works in isolation nor appears to be competing. Both reasoning and empathy associate positively with altruistic equity and effectiveness. That is, a balance between affective and cognitive capacities appears to offer a more integrated and impactful approach to helping others than either capacity on its own.

## Materials and methods

### Open science practices

This article examines empathic ability, reasoning ability, and prosociality among Effective Altruists, Extraordinary Altruists, and general population controls. It is part of a larger project leveraging the same extensive dataset to address a variety of distinct research questions that will be presented in separate articles. Due to the uniqueness and rarity of the subject groups, data for all related projects were collected simultaneously to ensure efficiency and make the best use of our participants' time. Additional articles from this dataset will explore topics such as moral beliefs and values, creative and imaginative capacity, close relationship quality, future-oriented thinking, and attitudes toward animals and nature within the same samples. Further details about the full dataset and the broader project are available in a “Read Me” file on the Open Science Framework (OSF) at https://osf.io/gsr8x/?view_only=d8773d7723404e36834782b945e4f46f. The OSF also houses additional relevant materials, including the raw dataset (featuring all measured variables across the related projects), the cleaned dataset containing the variables specific to this article, the full surveys administered to each sample, and analysis scripts related to the current article.

Sample characteristics, as well as the hypotheses and analyses for both within-group and between-group comparisons, were pre-registered on aspredicted.org. Pre-registrations for within-group analyses (e.g. relationships between empathic ability, reasoning ability, and prosociality) are available for the Effective Altruist (EA) sample (https://aspredicted.org/ry48-3wjp.pdf), the Extraordinary Altruist (XA) sample (https://aspredicted.org/y8mp-7r8t.pdf), and the control sample (https://aspredicted.org/qnnh-8ssq.pdf). Pre-registrations for between-group analyses (e.g. comparisons across EA, XA, and control groups on key measures) can be found at https://aspredicted.org/xfsx-7dmd.pdf. Additional recruitment criteria specific to the demographic matching of controls to their target groups were pre-registered at https://aspredicted.org/czjn-yb3p.pdf. Since EA recruitment relied on social media, Slack channels, and forums, further exclusion criteria to identify and exclude fraudulent responses (i.e. survey bots) were pre-registered at https://aspredicted.org/vjnk-yhgb.pdf. Data from responses excluded based on these criteria are publicly accessible on the OSF.

### Participants

An a priori power analysis determined that a sample of *N* = 319 subjects per participant group would provide 95% power to detect an effect size of *r* = 0.2 (two-tailed test, α = 0.05). Given the specialized nature of the two special population samples, we planned to collect as many cases as possible within 90 days of active data collection or until *N* = 319 was reached, whichever came first.

#### Sample 1: Effective altruists

We recruited effective altruists (EAs) using social media channels associated with the EA movement [e.g. The “Giving What We Can” Slack Channel, The Effective Altruism Forum, the Twitter (X) account of Peter Singer, a prominent figure within the EA movement]. Participants who self-identified as EAs were directed to complete a survey on Qualtrics, receiving $15 for their participation. After accounting for exclusions made on the basis of our pre-registered criteria for fraudulent responding (e.g. nonsensical or duplicate open-ended responses), 206 self-identifying EAs completed the survey during the 90 days of active recruitment. Of the 206 respondents, 87 were excluded for failing attention checks or possessing duplicate IP addresses, leaving *N* = 119 EAs in sample 1. Sensitivity analysis indicated 95% power to detect *r* = 0.32 or 80% power to detect *r* = 0.25 (two-tailed test, *α* = 0.05).

#### Sample 2: Extraordinary altruists

Extraordinary altruists (i.e. XAs–nondirected kidney, liver, and tissue donors) were recruited through an existing network of XAs maintained by a research laboratory at a prominent university in the Eastern United States. Participants who had donated an organ to a stranger completed the survey on Qualtrics and received $15. Of the 66 respondents who completed the survey, only one was excluded for failing attention checks, resulting in *N* = 65 donors (57 kidney donors, 7 kidney/liver double-donors, 1 kidney/marrow double-donor). This sample achieved 95% power to detect *r* = 0.43 or 80% power to detect *r* = 0.34 (two-tailed test, *α* = 0.05). Though a small sample, this sample size aligns with similar research on nondirected living donors and reflects the rarity of such costly altruism (24, 91, 92).

#### Sample 3: General population controls

The control sample consisted of English-speaking general population participants who (i) did not identify as effective altruists and (ii) had not donated an organ or body tissue to a stranger. The target sample size was designed to match the combined size of the two special population samples (samples 1 and 2). We recruited control participants via Prolific in two phases, matching demographics to sample 1 (EAs) and sample 2 (XAs) respectively. In phase 1, we aimed to recruit *N* = 119 controls matched to sample 1 on nationality, gender, and age. To account for exclusions, *N* = 139 participants were recruited. One additional participant completed the survey without submitting for remuneration and was retained in the raw dataset. After excluding 21 participants who failed more than three of the 13 attention checks, data from *N* = 109 participants were retained.

In phase 2, we aimed to recruit *N* = 65 controls matched to sample 2 on nationality, gender, age, and race/ethnicity. For the phase 2 controls, Prolific's quota sampling functionality enabled us to include the additional dimension of race/ethnicity in the matching criteria due to the smaller number of nationalities represented in the XA sample. To allow for exclusions, *N* = 75 participants were recruited. Again, one additional participant completed the survey without submitting for remuneration and was retained in the raw dataset. Nine participants were excluded for failing attention checks, leaving *N* = 67 participants in this phase. Across both phases, no participants had duplicate IP addresses. In total, the control sample comprised *N* = 176 participants, achieving 95% power to detect *r* = 0.27 or 80% power to detect *r* = 0.21 (two-tailed test, *α* = 0.05).

#### Combined sample

Across all samples, data were collected from *N* = 360 participants (*N* = 119 EAs, *N* = 65 donors, *N* = 176 controls), achieving 95% power to detect *r* = 0.19 or 80% power to detect *r* = 0.15 (two-tailed test, *α* = 0.05). Table S1 provides demographic details for each sample.

### Materials and procedure

This research includes data from human participants and the procedures were approved by the University at Albany, SUNY IRB (Protocol Number: 22X187). After providing informed consent electronically, participants completed a Qualtrics survey containing well-validated measures (93–100) assessing the predictors (i.e. empathic ability, reasoning ability) and outcomes (i.e. equitable and effective prosociality) in a randomized order. Demographic data and debriefing followed. Payment details were collected separately to protect anonymity, with samples 1 and 2 receiving $15 gift cards via email and sample 3 paid directly through Prolific. Table S2 comprehensively covers key information for each of these measures in detail, including example items, reliability statistics, scoring procedures, and scale interpretation. The full text of these items can be located with the full surveys on the OSF page.

## Supplementary Material

pgag015_Supplementary_Data

## Data Availability

Details about the full dataset and the broader project are available in a “Read Me” file on the Open Science Framework (OSF) at https://osf.io/gsr8x/?view_only=a0661bbcb3f84b2aa63ca3ac8504d792. The OSF also houses additional relevant materials, including the raw dataset (featuring all measured variables across the related projects), the cleaned dataset containing the variables specific to this article, the full surveys administered to each sample, and analysis scripts related to the current article.

## Notes

a The measures capturing engagement in real-world charitable actions were included as part of the demographics survey, and analyses pertaining to these measures were not pre-registered.

b Despite not reaching significance, this effect had a *Cohen’s d* of 0.315, which is greater than negligible. Nonetheless, further research with a larger sample size is required to elucidate whether this effect is robust or more likely to be an artifact. As such, these findings are inconclusive and should be interpreted with caution.

## Preprints

This manuscript was posted as a preprint: https://osf.io/preprints/psyarxiv/jm8gk_v4.
