# Supplementary material for: Reasoning and empathy are not competing but complementary features of altruism
Source: PNAS Nexus. 2026 Jan 20;5(2):pgag015. doi: 10.1093/pnasnexus/pgag015 (PMC12887898; doi:10.1093/pnasnexus/pgag015)
Supplement: pgag015_Supplementary_Data [file pgag015_supplementary_data.pdf]

Reasoning and empathy are not competing but complementary features of altruism

Supplementary Materials

Table of Contents

Sample Information..page 3

Key Detail on Measures...page 4

Sample Differences on Prosociality Measures..page 6

Sample Differences in Empathic and Reasoning Ability...page 9

Correlation Matrices......……………...…………………………………………...............page 11

Dimension Reduction.....……………...……………………………………..……..…............page 14

Prosociality Regressed on all Empathy and Reasoning Predictors Simultaneously.............page 16

Prosociality Regressed on Empathy and Reasoning Composites...page 21

Prosociality Regressed on the Interaction of Empathy and Reasoning................................page 23

Supplementary References............…………………………………………………….............page 25

Survey Instrument……………………………………………………………………….page 26

Sample Information

Table S1

Information about the three samples

Parameter		Sample 1: Effective Altruists (EAs)	Sample 2: Extraordinary Altruists (XAs)	Sample 3: Controls
Information About the Populations	Population Description	Individuals who self-identify with the effective altruism movement	Non-directed living organ donors	Ordinary adults
	Population Characteristics	EAs seek to maximize the impact of their altruistic actions through charitable donations that help strangers beyond their immediate community	XAs have voluntarily donated an organ—while living—to a complete stranger they have never met	Controls were recruited using quota sampling to be demographically similar to Samples 1 and 2
	Scale of Altruism	EAs, on average, reported donating 15.6% of their yearly income to charitable causes	XAs comprised 57 kidney, 7 kidney/liver, and 1 kidney/bone marrow donor(s)	Controls, on average, reported donating only 3.7% of their yearly income to charitable causes
	Recruitment Channels	EA slack channels, forums, and social media groups	A validated research database of living organ donors	Prolific
Gender	N_{Total}	119	65	176
	N_{Male}	79	22	97
	N_{Female}	36	42	77
	N_{Other}	4	1	2
Race	N_{White}	73	59	117
	N_{Black}	17	0	22
	N_{Asian}	13	3	19
	$N_{Multiracial}$	8	2	11
	$N_{OtherRace}$	8	1	7
Geographic Region	$N_{UnitedStates}$	46	61	103
	N_{Canada}	2	4	8
	$N_{UnitedKingdom}$	13	0	17
	$N_{AfricanContinent}$	21	0	13
	$N_{AsianContinent}$	8	0	8
	$N_{EuropeanContinent}$	21	0	19
	$N_{SAmericanContinent}$	1	0	1
	$N_{Australia\&NZ}$	7	0	7
Age (years)	M_{age}	31.9	53.0	37.2
	SD_{age}	8.8	12.5	12.1
Income[1]	M_{income}	3.0	4.5	3.0
	SD_{income}	1.9	1.5	1.6
Education[2]	$M_{education}$	7.9	8.0	6.9
	$SD_{education}$	1.9	1.8	2.0

Note. [1]Income was captured on a 1 "less than $25,000" – 6 "$150,000 or more" scale. On average, EAs and Controls fell into the "$50,000 – $74,999" bracket, while XAs fell into the "$75,000 – $100,000" bracket. [2]Education was captured on a 1 "no schooling completed" – 11 "doctorate degree" scale. On average, EAs and XAs obtained a bachelor's degree or equivalent, while controls obtained an associate's degree or equivalent.

Key Detail on Measures

Table S2

Key information on the measures capturing empathic ability, reasoning ability, and equitable and effective prosociality

Empathic Ability (Predictor)	
Measure, Example Item, Reliability	High Score Interpretation, Scale Points
IRI–Empathic Concern IRI–EC[37]; 7 items (e.g., "I am often quite touched by things that I see happen"; α=0.86)	Greater empathy for the suffering of others (1-7 Likert)
Emotionally Evocative Statements Task EEST[67]; 100 statements (e.g., Anger: "Don't you have any real friends?"; Disgust: "I haven't showered in days."; Fear: "I want to punch you."; Sadness: "I forgot your birthday."; Happiness: "You're amazing.")	Greater ability to correctly identify emotions conveyed in statements (Scored 0-100: The number of correctly identified emotions)
Toronto Alexithymia Scale (Reverse Coded) TAS[68]; 20 items (e.g., "I am often confused about what emotion I am feeling."; α=0.86)	Greater ability to recognize one's own emotions (1-7 Likert)
Levenson Self-Report Psychopathy Scale (Reverse Coded) LSRP[69]; Primary Psychopathy: 16 items (e.g., "Making a lot of money is my most important goal."; α=0.88); Secondary Psychopathy: 10 items (e.g., "I have been in a lot of shouting matches with other people."; α=0.76)	Lower primary and secondary psychopathy or Greater emotional ability (1-7 Likert)
Theories of Empathy Scale TES[58]; 6 items (e.g., "Anybody can change how empathic a person they are."; α=0.91)	Greater beliefs in the malleability of empathy (1-7 Likert)
Parochial Empathy Scale–Outgroup Empathy PES[38]; 4 items (e.g., "Anybody can change how empathic a person they are."; α=0.75)	Greater empathy for distant targets (1-7 Likert)
Reasoning Ability (Predictor)	
Measure, Example Item, Reliability	High Score Interpretation, Scale Points
Need for Cognition NFC[70]; 18 items (e.g., "I would prefer complex to simple problems."; α=0.90)	Greater enjoyment from effortful cognitive endeavors (1-7 Likert)
Cognitive Reflection Test CRT[71,72]; 7 word problems (e.g., "A bat and ball cost $1.10 in total. The bat costs $1 more than the ball. How much does the ball cost?", Intuitive Answer: $0.10, Correct answer: $0.05)	Greater tendency to override intuition and engage deliberative reasoning on word problems (Scored 1-7: The number of correctly answered problems)
Heuristics and Bias Tasks HBT[73]; 5 word problems (e.g., "Imagine that we are tossing a fair coin...it has just come up heads 5 times in a row. For the 6th toss do you think that...", Correct Answer: Heads and tails are equally probable on the sixth toss.)	Greater utilization of algorithmic reasoning versus heuristics on word problems (Scored 1-7: The number of correctly answered problems)
Rational Experiential Index–Reasoning Ability REI[74]; 10 items (e.g., "I have a logical mind."; α=0.89)	Greater self-reports of reasoning ability (1-7 Likert)
Actively Open-Minded Thinking Scale	Greater belief in changing one's mind on the basis of evidence (1-7 Likert)

AOT[75]; 11 items (e.g., "People should take into consideration evidence which goes against conclusions they favor."; α=0.85)	
Measures of Equitable and Effective Prosociality (Outcome)	
Measure, Example Item, Reliability	High Score Interpretation, Scale Points
**Moral Judgment Vignettes**<br>MJV[9]; 8 short vignettes (e.g., "To what extent was it morally acceptable for the person in the story to donate money to the UN charity for people in another country instead of 1 friend?"; α=0.93	Greater moral acceptability of welfare-maximizing altruism directed towards distant beneficiaries (**equitable and effective altruism captured**; 1-9 Likert)
**Social Discounting Task**<br>SDT[76]; 27 hypothetical decisions ("Would you prefer $80 for yourself or $85 dollars for [a mutual friend or acquaintance]")	Greater preference for larger rewards for distant others over smaller rewards for oneself (**equitable and effective altruism captured**; Scored 0-27: The number of choices favoring a target other than oneself)
**Behavioral Donation Task**<br>BDT; Developed for the current research; 16 consequential choices between lifesaving (e.g., The Malaria Consortium) and life-improving (e.g., National Caregiving Foundation) charitable causes	Greater number of donations to effective vs. ineffective causes (**effective altruism captured**; Scored 0-16: The number of choices to donate actual resources [USD] to an effective versus ineffective cause)
**Effective Altruism Interest Scale**<br>EAIS[18]; Expansive Altruism: 6 items (e.g., "I am willing to make significant sacrifices for people in need that I don't know and will never meet."; α=0.87); Effectiveness Focus: 6 items (e.g., "It would be the right choice to refrain from helping one person if that makes it possible to help a larger number of people."; α=0.82	Greater endorsement of **effective** (effectiveness focus subscale) and **equitable** (expansive altruism subscale) altruism (1-7 Likert)
**Reported Real-World Charitable Action**<br>RWCA; Developed for the current research; Income: "In a given year, what percentage (out of 100) of your yearly income do you donate to charity?"; Time: "In a given year, what percentage (out of 100) of your time do you devote towards volunteering to help others?"	Greater proportions of income devoted to philanthropic causes (for income) and volunteerism (for time) in a given year (**general prosociality measured**; 0%-100% Slider)

Sample Differences on Prosociality Measures

Figure S1

Reported real-world charitable action among effective altruists, extraordinary altruists and general population controls

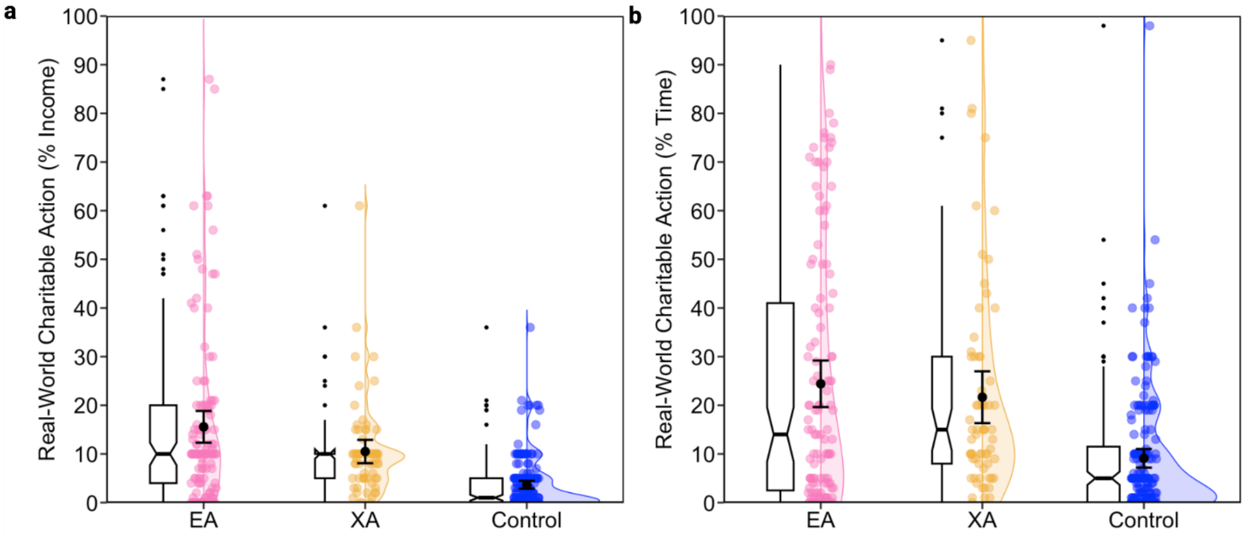

Note. Raincloud plots displaying the levels of financial (**a**) and temporal (**b**) resources devoted to the benefit of others by EAs, XAs and demographically-similar controls.

Figure S2

Differences between effective altruists, extraordinary altruists and general population controls on measures capturing equitable and effective altruism

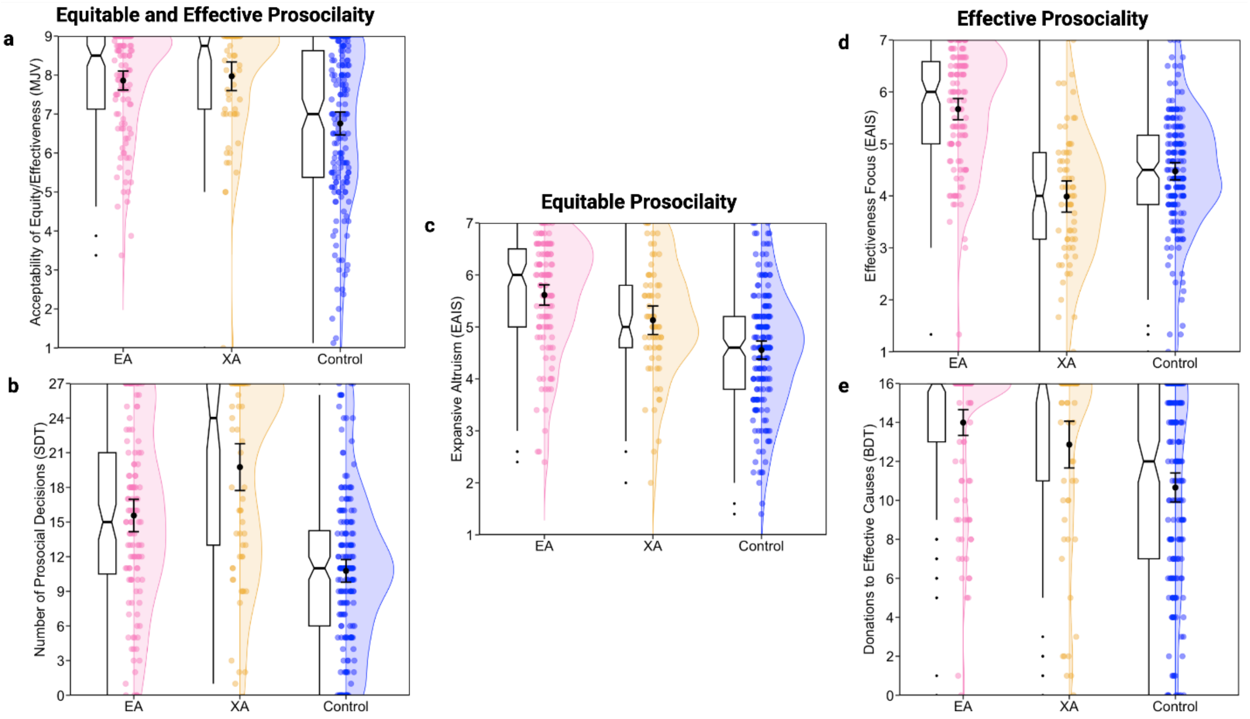

Note. Raincloud plots displaying differences between EAs, XAs and demographically-similar controls on metrics capturing (1) equitable and effective prosociality simultaneously: moral judgments in the MJV task (a) and the number of prosocial decisions made in the SDT (b); (2) equitable prosociality in isolation: explicit attitudes in alignment with expansive altruism on the EAIS (c); and (3) effective prosociality in isolation: explicit attitudes in alignment with endorsing effectiveness prioritization in altruistic decisions on the EAIS (d) and the number of donations made to effective causes vs. ineffective causes on the BDT (e).

Table S3

Results from one-way ANOVAs with Bonferroni-corrected post-hoc tests comparing Samples 1-3 on measures capturing prosociality

Sample	Prosociality
**Percentage of Income Donated (RWCA, Overall Prosociality)[1]**	
Omnibus	$F(2, 357) = 37.2, p < .001, \eta^2_p = .172$
EA vs. Control	$t(357) = 8.53,\ p < .001$, *Cohen's d* = 1.013
XA vs. Control	$t(357) = 4.00,\ p < .001$, *Cohen's d* = 0.580
EA vs. XA	$t(357) = 2.80,\ p = .016$, *Cohen's d* = 0.432
**Percentage of Time Spent Volunteering (RWCA, Overall Prosociality)[1]**	
Omnibus	$F(2, 357) = 23.9, p < .001, \eta^2_p = .118$
EA vs. Control	$t(357) = 6.51,\ p < .001$, *Cohen's d* = 0.772
XA vs. Control	$t(357) = 4.36,\ p < .001$, *Cohen's d* = 0.633
EA vs. XA	$t(357) = 0.90,\ p > .999$, *Cohen's d* = 0.139
**Moral Judgment Vignettes (MJV, Equitable & Effective Prosociality)**	
Omnibus	$F(2, 357) = 20.20, p < .001, \eta^2_p = 0.102$
EA vs. Control	$t(357) = 5.45,\ p < .001$, *Cohen's d* = 0.647
XA vs. Control	$t(357) = 4.90,\ p < .001$, *Cohen's d* = 0.711
EA vs. XA	$t(357) = -0.42,\ p > .999$, *Cohen's d* = -0.065
**Social Discounting (SDT, Equitable & Effective Prosociality)**	
Omnibus	$F(2, 357) = 40.40, p < .001, \eta^2_p = 0.185$
EA vs. Control	$t(357) = 5.55,\ p < .001$, *Cohen's d* = 0.659
XA vs. Control	$t(357) = 8.51,\ p < .001$, *Cohen's d* = 1.235
EA vs. XA	$t(357) = -3.73,\ p < .001$, *Cohen's d* = -0.576
**Expansive Altruism (EAIS, Equitable Prosociality)**	
Omnibus	$F(2, 357) = 31.80, p < .001, \eta^2_p = .151$
EA vs. Control	$t(357) = 7.92,\ p < .001$, *Cohen's d* = 0.940
XA vs. Control	$t(357) = 3.51,\ p = .002$, *Cohen's d* = 0.509
EA vs. XA	$t(357) = 2.79,\ p = .017$, *Cohen's d* = 0.431
**Effectiveness Focus (EAIS, Effective Prosociality)**	
Omnibus	$F(2, 357) = 59.30, p < .001, \eta^2_p = .249$
EA vs. Control	$t(357) = 8.92,\ p < .001$, *Cohen's d* = 1.058
XA vs. Control	$t(357) = -2.96,\ p = .010$, *Cohen's d* = -0.429
EA vs. XA	$t(357) = 9.64,\ p < .001$, *Cohen's d* = 1.488
**Behavioral Donations to Effective Causes (BDT, Effective Prosociality)**	
Omnibus	$F(2, 357) = 19.8, p < .001, \eta^2_p = .100$
EA vs. Control	$t(357) = 6.14,\ p < .001$, *Cohen's d* = 0.729
XA vs. Control	$t(357) = 3.32,\ p = .003$, *Cohen's d* = 0.482
EA vs. XA	$t(357) = 1.60,\ p = .329$, *Cohen's d* = 0.247

Note. [1]These models were estimated without controlling for variation in income. To rule out the possibility that the observed Sample effects on real-world charitable action owe to differences in income between the three samples, exploratory, non-preregistered models controlling for income can be found in the analysis script posted on OSF. In short, these exploratory models revealed the same patterns as those presented in the main text.

Sample Differences in Empathic and Reasoning Ability

Table S4

Results from one-way ANOVAs with Bonferroni-corrected post-hoc tests comparing Samples 1-3 on measures of empathic and reasoning ability

**Sample**	**Empathic Ability**
**Empathic Concern (IRI–EC)**[1]	
Omnibus	$F(2, 357) = 6.34$, $p = .002$, $\eta^2p = .034$
EA vs. Control	$t(357) = -0.69$, $p > .999$, *Cohen's d* = -0.082
XA vs. Control	$t(357) = 3.07$, $p = .007$, *Cohen's d* = 0.445
EA vs. XA	$t(357) = -3.42$, $p = .002$, *Cohen's d* = -0.527
**Ability to Identify Emotions in Statements (EEST)**	
Omnibus	$F(2, 357) = 18.70$, $p < .001$, $\eta^2p = .095$
EA vs. Control	$t(357) = -5.82$, $p < .001$, *Cohen's d* = -0.691
XA vs. Control	$t(357) = -0.17$, $p > .999$, *Cohen's d* = -0.025
EA vs. XA	$t(357) = -4.32$, $p < .001$, *Cohen's d* = -0.666
**Emotional Awareness (TAS)**	
Omnibus	$F(2, 357) = 8.22$, $p < .001$, $\eta^2p = .044$
EA vs. Control	$t(357) = -1.14$, $p = .762$, *Cohen's d* = -0.136
XA vs. Control	$t(357) = 3.30$, $p = .003$, *Cohen's d* = 0.479
EA vs. XA	$t(357) = -3.98$, $p < .001$, *Cohen's d* = -0.614
**Reverse-Coded Primary Psychopathy (LSRP Primary Psychopathy)**	
Omnibus	$F(2, 357) = 24.00$, $p < .001$, $\eta^2p = .118$
EA vs. Control	$t(357) = 0.92$, $p > .999$, *Cohen's d* = 0.109
XA vs. Control	$t(357) = 6.78$, $p < .001$, *Cohen's d* = 0.984
EA vs. XA	$t(357) = -5.67$, $p < .001$, *Cohen's d* = -0.875
**Reverse-Coded Secondary Psychopathy (LSRP Secondary Psychopathy)**	
Omnibus	$F(2, 357) = 16.40$, $p < .001$, $\eta^2p = .084$
EA vs. Control	$t(357) = -0.90$, $p > .999$, *Cohen's d* = -0.107
XA vs. Control	$t(357) = 5.03$, $p < .001$, *Cohen's d* = 0.731
EA vs. XA	$t(357) = -5.43$, $p < .001$, *Cohen's d* = -0.838
**Beliefs that Empathy is Malleable (TES)**	
Omnibus	$F(2, 357) = 0.72$, $p = .486$, $\eta^2p = .004$
EA vs. Control	$t(357) = 0.66$, $p > .999$, *Cohen's d* = 0.078
XA vs. Control	$t(357) = 1.17$, $p = .731$, *Cohen's d* = 0.170
EA vs. XA	$t(357) = -0.59$, $p > .999$, *Cohen's d* = -0.092
**Outgroup Empathy (PES)**[2]	
Omnibus	$F(2, 357) = 2.67$, $p = .071$, $\eta^2p = .015$
EA vs. Control	$t(357) = 1.46$, $p = .433$, *Cohen's d* = 0.174
XA vs. Control	$t(357) = 2.17$, $p = .092$, *Cohen's d* = 0.315
EA vs. XA	$t(357) = -0.92$, $p > .999$, *Cohen's d* = -0.141
**Sample**	**Reasoning Ability**
**Need for Cognition (NFC)**	
Omnibus	$F(2, 357) = 10.20$, $p < .001$, $\eta^2p = .054$
EA vs. Control	$t(357) = 4.51$, $p < .001$, *Cohen's d* = 0.536
XA vs. Control	$t(357) = 1.49$, $p = .414$, *Cohen's d* = 0.216
EA vs. XA	$t(357) = 2.07$, $p = .116$, *Cohen's d* = 0.320
**Ability to Override Intuition on Word Problems (CRT)**	
Omnibus	$F(2, 357) = 19.40$, $p < .001$, $\eta^2p = .098$
EA vs. Control	$t(357) = 4.34$, $p < .001$, *Cohen's d* = 0.515
XA vs. Control	$t(357) = -2.78$, $p = .017$, *Cohen's d* = -0.403

EA vs. XA	$t(357) = 5.96$, $p < .001$, *Cohen's d* = 0.919
**Tendency to Use Algorithmic vs. Heuristic Thinking on Word Problems (HBT)**	
Omnibus	$F(2, 357) = 10.80$, $p < .001$, $\eta^2p = .057$
EA vs. Control	$t(357) = 4.37$, $p < .001$, *Cohen's d* = 0.520
XA vs. Control	$t(357) = 2.91$, $p = .012$, *Cohen's d* = 0.422
EA vs. XA	$t(357) = 0.63$, $p > .999$, *Cohen's d* = 0.098
**Self-Reported Reasoning Ability (REI)**	
Omnibus	$F(2, 357) = 0.08$, $p = .920$, $\eta^2p < .001$
EA vs. Control	$t(357) = -0.41$, $p > .999$, *Cohen's d* = -0.048
XA vs. Control	$t(357) = -0.16$, $p > .999$, *Cohen's d* = -0.023
EA vs. XA	$t(357) = -0.16$, $p > .999$, *Cohen's d* = -0.025
**Actively Open-Minded Thinking (AOT)**	
Omnibus	$F(2, 357) = 2.22$, $p = .110$, $\eta^2p = .012$
EA vs. Control	$t(357) = 0.81$, $p > .999$, *Cohen's d* = 0.096
XA vs. Control	$t(357) = 2.11$, $p = .108$, *Cohen's d* = 0.306
EA vs. XA	$t(357) = -1.36$, $p = .522$, *Cohen's d* = -0.210

Note. [1]Although secondary to the primary focus of the investigation, we included the "Personal Distress" subscale of the IRI, pre-registering that we would evaluate sample differences on this measure. An omnibus effect of sample was observed, $F(2, 357) = 17.59$, $p < .001$, $\eta^2p = .065$. Intriguingly, XAs scored lower than both Controls ($t(357) = -4.65$, $p < .001$, *Cohen's d* = -0.675) and EAs ($t(357) = -4.53$, $p < .001$, *Cohen's d* = -0.698), while Controls and EAs did not differ from one another, $t(357) = -0.20$, $p > .999$, *Cohen's d* = -0.023. These findings further suggest that XAs show enrichments in empathic ability–feeling concern for the welfare others without becoming personally distressed. Personal distress was negatively associated with some measures of prosociality (see the SOM for details). [2]We additionally included the ingroup empathy subscale of the PES, but did not have specific predictions regarding group differences. The omnibus test was non-significant, $F(2, 357) = 2.98$, $p = .052$, $\eta^2p = .016$. Interestingly, however, ingroup empathy had significant positive associations with some measures of prosociality among EAs (see the SOM for details).

Correlation Matrices

EA Sample

See Table S5 for correlations among measures of prosociality, empathic ability, and reasoning ability for the EA sample.

Table S5

Correlations among measures of prosociality, empathic ability, and reasoning ability for the EA sample

	1	2	3	4	5	6	7	8	9	10	11	12	13	14	15	16	17	18	19	20
1. MJV	—																			
2. SDT	0.175	—																		
3. EAIS-EX	0.535***	0.228*	—																	
4. EAIS-EF	0.692***	0.157	0.632***	—																
5. BDT	0.493***	0.117	0.498***	0.495***	—															
6. RWCA-Money	-0.268**	0.141	-0.111	-0.239**	-0.147	—														
7. RWCA Time	-0.365***	0.124	-0.109	-0.283**	-0.195*	0.494***	—													
8. IRI-EC	0.322***	0.265**	0.578***	0.299***	0.413***	0.012	0.107	—												
9. EEST	0.51***	-0.038	0.339***	0.339***	0.682***	-0.219*	-0.287**	0.333***	—											
10. TAS	0.401***	0.108	0.237**	0.288**	0.316***	-0.031	-0.211*	0.275**	0.465***	—										
11. LSRP-P	0.535***	0.276**	0.433***	0.368***	0.558***	-0.129	-0.219*	0.57***	0.666***	0.555***	—									
12. LSRP-S	0.384***	0.262**	0.309***	0.279**	0.429***	-0.06	-0.114	0.379***	0.492***	0.65***	0.588***	—								
13. TES	0.107	0.171	0.155	0.074	0.095	0.036	0.169	0.338***	-0.009	0.044	0.202*	0.156	—							
14. PES (Outgroup)	0.279**	0.125	0.46***	0.364***	0.219*	-0.007	0.074	0.489***	0.095	-0.052	0.226*	0.087	0.234*	—						
15. NFC	0.356***	-0.001	0.297**	0.339***	0.384***	-0.116	-0.186*	0.193*	0.475***	0.318***	0.408***	0.261**	0.046	0.011	—					
16. CRT	0.105	-0.031	-0.029	0.132	0.005	0.003	-0.343***	-0.229*	0.049	0.105	0.045	-0.07	-0.278**	-0.254**	0.191*	—				
17. HBT	0.412***	-0.044	0.296**	0.336***	0.462***	-0.25**	-0.411***	-0.019	0.592***	0.408***	0.434***	0.193*	-0.14	-0.079	0.429***	0.401***	—			
18. REI	0.473***	0.095	0.331***	0.498***	0.453***	-0.115	-0.139	0.181*	0.424***	0.342***	0.327***	0.383***	0.061	0.236**	0.641***	0.176	0.384***	—		
19. AOT	0.605***	0.096	0.415***	0.465***	0.557***	-0.292**	-0.397***	0.36***	0.701***	0.501***	0.746***	0.402***	0.056	0.135	0.602***	0.23*	0.665***	0.475***	—	
20. IRI-PD[1]	-0.294**	-0.141	-0.239**	-0.251**	-0.162	0.083	0.164	-0.008	-0.204*	-0.446***	-0.21*	-0.35***	-0.073	0.024	-0.23*	-0.12	-0.25**	-0.25**	-0.17	—
21. PES (Ingroup)[2]	0.269**	0.089	0.391***	0.217*	0.321***	-0.052	0.044	0.554***	0.21*	0.04	0.365***	0.205*	0.247**	0.728***	0.046	-0.3**	-0.06	0.226*	0.193*	0.154

Note. * p < .05, ** p < .01, *** p < .001. [1]The personal distress subscale of the IRI, which measures reported feelings of discomfort in response to the suffering of others, was unintentionally included in the key dependent variables section of the pre-registration, though it was originally intended as a filler scale. In keeping with our commitment to transparency, we are nonetheless reporting the variable's associations here. The findings corroborate earlier ones demonstrating that personal distress is negatively associated with empathy and prosociality[1]. [2]We additionally included the ingroup empathy subscale of the PES, but did not have specific predictions regarding its associations with measures of prosociality. Intriguingly, we observed positive associations with scores on the MJV, the expansive altruism and effectiveness focus subscales of the EAIS, as well as the BDT. These unexpected findings corroborate the broader pattern of results, suggesting that empathy, even for those who are close, may serve as a launching point for altruistic equity and effectiveness.

XA Sample

See Table S6 for correlations among measures of prosociality, empathic ability, and reasoning ability for the XA sample.

Table S6

Correlations among measures of prosociality, empathic ability, and reasoning ability for the XA sample

	1	2	3	4	5	6	7	8	9	10	11	12	13	14	15	16	17	18	19	20
1. MJV	—																			
2. SDT	0.124	—																		
3. EAIS-EX	0.163	0.243	—																	
4. EAIS-EF	0.101	-0.036	0.099	—																
5. BDT	0.328**	-0.041	0.387**	0.21	—															
6. RWCA-Money	0.101	0.137	0.124	0.096	-0.011	—														
7. RWCA Time	-0.096	0.141	0.006	-0.043	-0.059	0.252*	—													
8. IRI-EC	0.155	0.125	0.308*	0.019	0.156	0.154	0.098	—												
9. EEST	-0.058	0.129	-0.102	0.038	0.05	-0.248*	0.086	0.047	—											
10. TAS	0.187	0.021	0.273*	0.032	0.248*	0.035	0.055	0.278*	0.116	—										
11. LSRP-P	0.222	0.166	0.344**	0.087	0.38**	0.129	0.127	0.46***	0.09	0.329**	—									
12. LSRP-S	0.261*	0.125	0.176	0.114	0.407***	0.128	0.12	0.273*	0.144	0.604***	0.473***	—								
13. TES	0.03	0.168	0.23	-0.249*	-0.094	0.141	-0.017	0.067	-0.157	-0.047	0.09	-0.09	—							
14. PES (Outgroup)	0.052	0.369**	0.312*	0.007	-0.074	0.41***	0.273*	0.507***	-0.087	0.206	0.124	0.179	0.235	—						
15. NFC	0.199	0.133	0.307*	0.171	0.215	0.201	-0.011	-0.059	0.085	0.335**	0.123	0.233	0.117	0.106	—					
16. CRT	0.005	0.143	0.017	0.023	0.229	-0.115	-0.173	-0.144	0.227	-0.083	0.029	-0.07	0.223	-0.279*	0.252*	—				
17. HBT	0.206	0.084	0.133	0.273*	0.308*	-0.142	-0.179	-0.236	0.226	0.102	0.113	0.061	-0.187	-0.156	0.252*	0.248*	—			
18. REI	-0.014	0.13	0.118	0.162	0.053	-0.248*	0.003	0.049	0.306*	0.25*	0.049	0.158	-0.027	-0.093	0.384**	0.236	0.275*	—		
19. AOT	0.281*	0.112	0.154	0.122	0.46***	-0.034	-0.268*	0.028	0.16	0.195	0.492***	0.257*	0.134	-0.26*	0.363**	0.48***	0.384**	0.095	—	
20. IRI-PD	-0.171	-0.139	-0.198	-0.156	-0.151	-0.062	-0.227	-0.15	-0.031	-0.354**	-0.24	-0.3*	-0.017	-0.173	-0.35**	-0.18	-0.02	-0.48***	-0.05	—
21. PES (Ingroup)	0.027	0.119	0.153	-0.038	-0.008	0.306*	0.138	0.54***	-0.075	0.146	0.158	0.077	0.177	0.664***	-0.08	-0.33**	-0.23	-0.33**	-0.06	-0.02

Note. * p < .05, ** p < .01, *** p < .001. [1]The personal distress subscale of the IRI, which measures reported feelings of discomfort in response to the suffering of others, was unintentionally included in the key dependent variables section of the pre-registration, though it was originally intended as a filler scale. In keeping with our commitment to transparency, we are nonetheless reporting the variable's associations here. The findings corroborate earlier ones demonstrating that personal distress is negatively associated with empathy and prosociality[1]. [2]We additionally included the ingroup empathy subscale of the PES, but did not have specific predictions regarding its associations with measures of prosociality. Intriguingly, we observed positive associations with real world engagement in philanthropy.

Control Sample

See Table S7 for correlations among measures of prosociality, empathic ability, and reasoning ability for the Control sample.

Table S7

Correlations among measures of prosociality, empathic ability, and reasoning ability for the Control sample

	1	2	3	4	5	6	7	8	9	10	11	12	13	14	15	16	17	18	19	20
1. MJV	—																			
2. SDT	0.044	—																		
3. EAIS-EX	0.121	0.203 **	—																	
4. EAIS-EF	0.144	0.108	0.267***	—																
5. BDT	0.321***	0.075	0.205**	0.071	—															
6. RWCA-Money	-0.053	0.207 **	0.225**	0.007	0.024	—														
7. RWCA Time	-0.04	0.164 *	0.2**	0.072	0.133	0.475***	—													
8. IRI-EC	0.014	0.263 ***	0.506***	0.031	0.134	0.269***	0.193*	—												
9. EEST	0.119	-0.033	-0.133	-0.084	0.046	-0.051	-0.102	0.099	—											
10. TAS	0.002	0.023	0.124	-0.043	-0.022	0.108	0.075	0.323***	0.238**	—										
11. LSRP-P	0.184*	0.168 *	0.222**	-0.081	0.132	0.002	-0.073	0.491***	0.282***	0.467***	—									
12. LSRP-S	0.046	0.077	0.092	0.044	0.035	0.105	0.086	0.366***	0.12	0.659***	0.539***	—								
13. TES	0.022	0.099	0.218**	-0.144	0.21**	0.154*	0.071	0.208**	-0.085	0.046	0.095	0.101	—							
14. PES (Outgroup)	0.045	0.214 **	0.525***	0.095	0.144	0.212**	0.194**	0.572***	-0.023	0.212**	0.241**	0.224**	0.223**	—						
15. NFC	0.083	0.119	0.173*	0.034	0.043	0.016	0.021	0.285***	0.025	0.313***	0.301***	0.393***	0.165*	0.266***	—					
16. CRT	0.191*	-0.089	0.063	-0.003	0.097	-0.061	-0.095	-0.057	0.083	-0.014	0.061	-0.04	-0.004	-0.112	0.186*	—				
17. HBT	0.16*	-0.066	-0.031	0.001	0.108	-0.118	-0.123	-0.17*	0.021	-0.016	0.13	-0.02	-0.067	-0.204**	0.213**	0.381***	—			
18. REI	0.144	-0.008	0.043	0.131	-0.016	0.009	0.089	0.213**	0.08	0.473***	0.245**	0.518***	0.025	0.149*	0.641***	0.091	0.084	—		
19. AOT	0.339***	-0.019	0.033	-0.013	0.267***	-0.18*	-0.23**	0.119	0.33***	0.209**	0.477***	0.213**	0.063	0.045	0.258***	0.268***	0.277***	0.229**	—	
20. IRI-PD	-0.131	0.061	-0.044	0.016	0.068	-0.053	-0.095	-0.111	0.008	-0.516***	-0.25***	-0.47***	-0.151*	-0.036	-0.39***	0.011	-0.11	-0.47***	-0.14	—
21. PES (Ingroup)	-0.038	0.186 *	0.364***	-0.059	0.098	0.228**	0.177*	0.585***	-0.097	0.192*	0.293***	0.277***	0.235**	0.596***	0.205**	-0.01	-0.03	0.214**	0.118	-0.023

Note. * p < .05, ** p < .01, *** p < .001. [1]The personal distress subscale of the IRI, which measures reported feelings of discomfort in response to the suffering of others, was unintentionally included in the key dependent variables section of the pre-registration, though it was originally intended as a filler scale. In keeping with our commitment to transparency, we are nonetheless reporting the variable's associations here. The findings corroborate earlier ones demonstrating that personal distress is negatively associated with empathy and prosociality[1]. [2]We additionally included the ingroup empathy subscale of the PES, but did not have specific predictions regarding its associations with measures of prosociality. Intriguingly, we observed positive associations with scores on the SDT, the expansive altruism subscale of the EAIS, and with real world engagement in philanthropy and volunteerism. These unexpected findings corroborate the broader pattern of results, suggesting that empathy, even for those who are close, may serve as a launching point for altruistic equity and effectiveness.

Dimension Reduction

Exploratory Factor Analysis

Prior to estimating regression models to assess the independent and interactive effects of empathic ability and reasoning ability on the battery of prosociality metrics, we conducted Exploratory Factor Analysis (EFA) using the maximum likelihood extraction method in combination with oblimin rotation on the battery of measures of empathic ability and reasoning ability, separately. For empathic ability, the measures loaded onto a single factor, but outgroup empathy on the PES and scores on the EEST had weak loadings, below 0.5, and thus were excluded from the next stage of analysis. Likewise, for reasoning ability, each measure loaded onto a single factor, but scores on the CRT had loadings below 0.5, and thus were excluded from the next stage of analysis. See Table S8 and Figures S3-S4 for the results from the EFAs.

Table S8

Factor loadings from EFA

Variable	Factor	
Empathic Ability	1	Uniqueness
LSRPC_S	0.784	0.385
LSRP_P	0.779	0.394
TAS	0.715	0.489
IRI-EC	0.586	0.657
EEST	0.446	0.801
PES-Outgroup	0.321	0.897
TES	0.975	
Variable	Factor	
Reasoning Ability	1	Uniqueness
NFC	0.751	0.436
REI	0.626	0.608
AOT	0.571	0.674
HBT	0.518	0.732
CRT	0.362	0.869

Figure S3

Scree plot for empathic ability

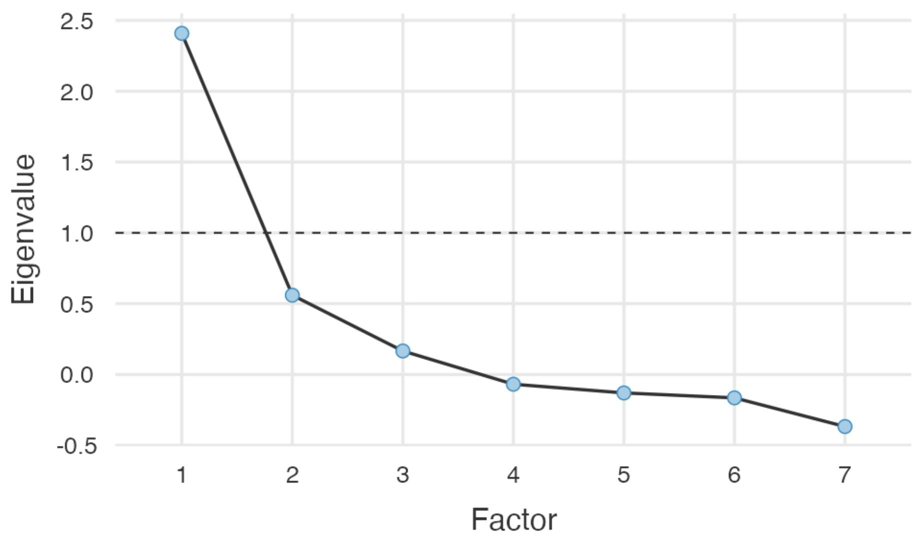

Figure S4

Scree plot for reasoning ability

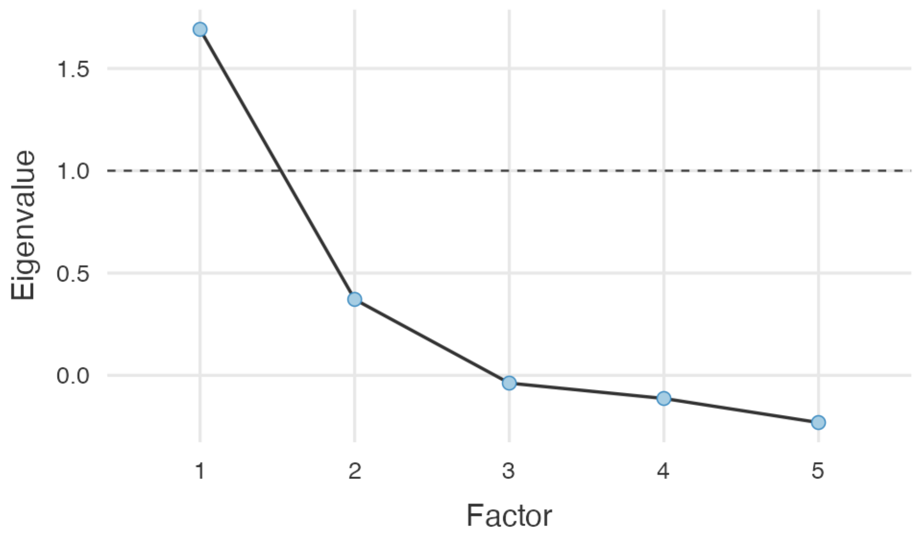

Multiple Regressions Including All Measures of Empathy and Reasoning as Simultaneous Predictors of Prosociality

We conducted a series of seven multiple regression models per sample (one per prosocial outcome, 21 in total), entering each measure of empathy and reasoning as a simultaneous predictor. The goal of estimating these models was to ascertain which predictors accounted for unique variance in the outcomes, above and beyond the effects of the other predictors. As pre-registered, because of the vast number of number of predictors included, we evaluated the variance inflation factor (VIF) for each predictor, which ranged from 1.11 to 2.34, indicating the predictors were only moderately correlated with one another and that multicollinearity was not problematic[2].

Within each sample, these results largely confirmed that both empathic and reasoning ability can be a force for good (see Table S9). Notably, empathic capacities were positively and significantly associated with: (1) generosity on the Social Discounting Task for EAs and XAs; (2) attitudes aligned with expansive altruism (altruistic equity) on the EAIS for EAs and controls; and intriguingly, with (3) attitudes aligned with effectiveness prioritization on the EAIS for EAs, (4) behavioral donations to effective charitable causes across all three samples, (5) real world monetary charitable contributions (as the percentage of income donated in a given year) for XAs and controls, and (6) with real-world time invested volunteering (as the percentage of time spent volunteering in a given year) among XAs. These findings suggest that among exceptional altruists and ordinary adults alike, greater empathy and emotionality often predicts greater attitudes, judgments, decisions and behaviors in line with altruistic equity, effectiveness, and real-world charitable action, above and beyond differences in reasoning ability. Critically, they provide clear evidence that arguments against empathy may be misguided[3,4], as greater ability to

empathize with others appears to associate positively with rather than inhibit boundary transcendent altruism and prosociality that maximizes welfare.

Table S9

Results from multiple linear regression models evaluating associations between individual variation in empathy and reasoning with individual variation in equitable, effective, and real-world prosociality within Samples 1, 2, and 3

Outcome Predictor	**EA** (R^2 = 0.47)		**XA** (R^2 = 0.20)		**Control** (R^2 = 0.16)	
MJV	*β*	*p*	*β*	*p*	*β*	*p*
IRI-EC	-0.013	.898	0.255	.168	-0.081	.424
EEST	0.103	.387	-0.116	.402	0.028	.727
TAS	0.080	.447	-0.030	.859	-0.154	.130
LSRP-P	0.072	.610	-0.087	.632	0.129	.234
LSRP-S	0.024	.832	0.214	.217	-0.028	.802
TES	0.036	.653	0.073	.621	0.020	.790
PES	0.159	.086	-0.105	.564	0.111	.223
NFC	-0.139	.199	0.157	.336	-0.142	.169
CRT	0.033	.700	-0.108	.508	0.105	.193
HBT	0.011	.924	0.255	.109	0.052	.542
REI	**0.237**	**.029**	-0.141	.367	**0.216**	**.043**
AOT	**0.368**	**.013**	0.158	.440	**0.254**	**.005**
Outcome Predictor	**EA** (R^2 = 0.23)		**XA** (R^2 = 0.28)		**Control** (R^2 = 0.11)	
SDT	*β*	*p*	*β*	*p*	*β*	*p*
IRI-EC	0.143	.261	-0.216	.218	0.166	.111
EEST	**-0.438**	**.003**	0.085	.518	-0.032	.694
TAS	-0.156	.218	-0.168	.292	-0.076	.466
LSRP-P	**0.426**	**.013**	0.126	.463	0.141	.207
LSRP-S	0.247	.067	0.067	.683	-0.009	.938
TES	0.021	.827	-0.006	.967	0.020	.801
PES	-0.061	.584	**0.603**	**< .001**	0.069	.464
NFC	-0.120	.357	-0.088	.567	0.143	.178
CRT	0.009	.932	0.155	.318	-0.071	.393
HBT	0.047	.740	-0.025	.869	-0.016	.852
REI	0.151	.247	0.184	.218	-0.114	.299
AOT	-0.013	.939	0.165	.396	-0.069	.460
Outcome Predictor	**EA** (R^2 = 0.50)		**XA** (R^2 = 0.33)		**Control** (R^2 = 0.40)	
**EAIS-EX**	*β*	*p*	*β*	*p*	*β*	*p*
IRI-EC	**0.492**	**< .001**	0.204	.229	**0.336**	**< .001**
EEST	-0.113	.324	-0.151	.235	**-0.171**	**.012**
TAS	-0.074	.466	0.112	.465	0.075	.381
LSRP-P	-0.089	.513	0.242	.148	0.065	.476
LSRP-S	0.152	.158	-0.079	.617	-0.139	.134
TES	-0.031	.683	0.171	.211	0.068	.284
PES	**0.264**	**.004**	0.096	.566	**0.349**	**< .001**
NFC	0.098	.350	0.241	.110	-0.004	.962

CRT	-0.005	.955	0.011	.941	0.115	.090
HBT	**0.374**	**.001**	0.212	.145	0.063	.378
REI	0.015	.889	-0.001	.993	-0.056	.535
AOT	0.012	.932	-0.120	.521	-0.022	.773
Outcome	**EA**		**XA**		**Control**	
Predictor	(R^2 = 0.40)		(R^2 = 0.17)		(R^2 = 0.09)	
**EAIS-EF**	*β*	*p*	*β*	*p*	*β*	*p*
IRI-EC	0.079	.482	0.046	.804	0.057	.587
EEST	-0.053	.673	-0.092	.513	-0.068	.410
TAS	0.057	.607	-0.165	.334	-0.153	.150
LSRP-P	-0.063	.676	0.022	.904	-0.156	.168
LSRP-S	0.042	.723	0.084	.632	0.126	.270
TES	-0.005	.948	-0.277	.072	**-0.175**	**.027**
PES	**0.274**	**.006**	0.101	.587	0.143	.133
NFC	-0.048	.678	0.120	.469	-0.092	.390
CRT	0.083	.371	-0.008	.960	0.004	.961
HBT	0.099	.424	0.190	.238	0.037	.677
REI	**0.288**	**.013**	0.114	476	0.195	.079
AOT	**0.245**	**.117**	0.075	.720	0.054	.565
Outcome	**EA**		**XA**		**Control**	
Predictor	(R^2 = 0.57)		(R^2 = 0.39)		(R^2 = 0.15)	
**BDT**	*β*	*p*	*β*	*p*	*β*	*P*
IRI-EC	**0.211**	**.028**	0.207	.203	0.086	.399
EEST	**0.423**	**< .001**	-0.119	.325	-0.002	.980
TAS	-0.174	.069	-0.028	.847	-0.105	.306
LSRP-P	0.054	.673	0.063	.692	-0.039	.720
LSRP-S	0.117	.247	**0.327**	**.034**	0.050	.651
TES	0.014	.843	-0.106	.415	**0.175**	**.023**
PES	-0.001	.994	-0.119	.457	0.116	.207
NFC	-0.065	.509	0.080	.574	-0.087	.404
CRT	-0.047	.544	0.173	.227	0.029	.720
HBT	**0.215**	**.042**	0.234	.093	0.099	.248
REI	**0.201**	**.041**	-0.133	.330	-0.039	.718
AOT	-0.005	.972	0.158	.379	**0.268**	**.004**
Outcome	**EA**		**XA**		**Control**	
Predictor	(R^2 = 0.15)		(R^2 = 0.33)		(R^2 = 0.15)	
**RWCA ($)**	*β*	*p*	*β*	*p*	*β*	*p*
IRI-EC	0.083	.533	-0.034	.842	**0.264**	**.010**
EEST	-0.034	.821	-0.163	.200	0.004	.964
TAS	0.179	.179	-0.134	.386	0.086	.404
LSRP-P	0.149	.403	0.120	.468	-0.125	.252
LSRP-S	-0.075	.596	0.113	.476	0.074	.506
TES	0.022	.828	-0.025	.852	0.108	.159
PES	0.033	.779	**0.338**	**.047**	0.068	.462
NFC	0.122	.373	**0.313**	**.039**	-0.050	.632
CRT	0.151	.169	0.039	.795	0.028	.728
HBT	-0.123	.402	-0.065	.651	0.022	.797
REI	-0.033	.806	-0.262	.071	-0.038	.725
AOT	**-0.484**	**.010**	-0.060	.750	**-0.188**	**.040**
Outcome	**EA**		**XA**		**Control**	
Predictor	(R^2 = 0.29)		(R^2 = 0.24)		(R^2 = 0.14)	
**RWCA (Time)**	*β*	*p*	*β*	*p*	*β*	*p*
IRI-EC	0.222	.070	-0.230	.204	0.190	.065
EEST	-0.075	.583	0.151	.267	-0.029	.722
TAS	-0.027	.823	-0.038	.815	0.036	.724

LSRP-P	0.045	.783	**0.383**	**.034**	-0.141	.199
LSRP-S	-0.068	.595	0.046	.784	0.060	.587
TES	0.061	.509	-0.063	.663	0.038	.623
PES	-0.076	.476	0.249	.167	0.109	.241
NFC	0.056	.657	0.062	.696	-0.090	.390
CRT	-0.203	.045	0.037	.816	-0.005	.950
HBT	-0.073	.590	-0.166	.283	0.025	.770
REI	0.104	.403	0.023	.883	0.125	.246
AOT	**-0.398**	**.020**	-0.384	.059	**-0.215**	**.020**

Note. Bolded effects indicate statistical significance at $p < .05$.

Furthermore, these results partially align with assertions raised in discourse related to the effective altruism movement[5] and with some earlier empirical findings[6] suggesting that reasoning ability underlies the prioritization of altruistic equity and effectiveness. Namely, variation in reasoning ability was significantly and positively associated with: (1) moral judgments of equitable and effective prosociality on the MJV task among EAs and controls; (2) the prioritization of expansiveness (equity) and effectiveness in altruism on the EAIS among EAs; (5) behavioral donations to effective causes among EAs and Controls; and (6) real-world monetary contributions to charity among XAs. However, it is noteworthy that the associations between reasoning ability with equitable and effective prosociality were most pronounced among members of the EA subject group, who explicitly emphasize applying reasoning skills to guide altruistic decision-making.

Collectively, these findings suggest that both empathy and reasoning, rather than one over the other, are generally associated with greater altruistic equity, effectiveness, and real-world charitable action. Importantly, empathy does not consistently constrain the scope of equity and impact. However, there are exceptions. For instance, broader emotional abilities, such as accurately identifying emotions in written statements (measured by the EEST), were negatively associated with generosity on the SDT among EAs and the prioritization of expansiveness in altruism among controls. These unexpected results warrant further investigation to clarify their implications.

It is also notable that controls who reported more malleable lay theories of empathy scored significantly lower in their prioritization of effectiveness in altruism. Prior research suggests that individuals with more malleable versus fixed theories of empathy often exert greater empathic effort toward distant and stigmatized targets[8]. Thus, this result was unexpected. One possibility is that it reflects an “empathic bystander effect”[9], where holding stronger beliefs about the malleability of empathy allows individuals to diffuse personal responsibility for addressing large-scale suffering. Because the measure captures beliefs about the malleability of empathy not only for oneself but also for others, individuals who score higher might overestimate others’ empathic capacities, assuming others will prioritize effectiveness in their stead. Further research is needed to explore this possibility.

Deviations from expected results were observed not only in measures of empathic ability but also in reasoning ability. For instance, Actively Open-Minded Thinking (AOT)—the willingness to change one’s mind when faced with new evidence—was moderately negatively associated with real-world charitable donations and volunteerism for EAs and weakly negatively associated for controls. These findings suggest that, in some contexts, reasoning abilities may backfire by increasing focus on the opportunity costs of giving. This effect may occur even among individuals who typically prioritize effectiveness in their altruistic efforts. However, these findings were exceptions to the general trend across samples and measures, where both reasoning ability and empathic ability were positively associated with equitable and effective prosocial attitudes and behaviors.

Multiple Regressions Including Empathy and Reasoning Composites as Simultaneous Predictors of Prosociality

The statistics presented below in Table S10 pertain to the results discussed in the main text, where the empathy and reasoning composites derived from factor analysis were treated as predictors of prosociality.

Table S10

Results from multiple linear regression models evaluating associations between individual variation in the empathic ability factor and the reasoning ability factor with individual variation in equitable, effective, and real-world prosociality within Samples 1, 2, and 3

Outcome	**EA**		**XA**		**Control**	
Predictor	(R^2 = 0.40)		(R^2 = 0.10)		(R^2 = 0.07)	
MJV	*β*	*p*	*β*	*p*	*β*	*p*
Empathic Ability	**0.290**	**0.001**	0.234	0.064	-0.050	0.547
Reasoning Ability	**0.423**	**< .001**	0.171	0.173	**0.277**	**< .001**
Outcome	**EA**		**XA**		**Control**	
Predictor	(R^2 = 0.10)		(R^2 = 0.04)		(R^2 = 0.08)	
SDT	*β*	*p*	*β*	*p*	*β*	*p*
Empathic Ability	**0.379**	**< .001**	0.108	0.406	**0.281**	**< .001**
Reasoning Ability	-0.163	0.125	0.142	0.275	-0.010	0.856
Outcome	**EA**		**XA**		**Control**	
Predictor	(R^2 = 0.27)		(R^2 = 0.16)		(R^2 = 0.10)	
**EAIS-EX**	*β*	*p*	*β*	*p*	*β*	*p*
Empathic Ability	**0.376**	**<.001**	**0.318**	**0.010**	**0.334**	**< .001**
Reasoning Ability	**0.213**	**0.027**	0.180	0.139	-0.062	0.447
Outcome	**EA**		**XA**		**Control**	
Predictor	(R^2 = 0.28)		(R^2 = 0.07)		(R^2 = 0.01)	
**EAIS-EF**	*β*	*p*	*β*	*p*	*β*	*p*
Empathic Ability	0.161	0.092	0.018	0.890	-0.052	0.544
Reasoning Ability	**0.420**	**<.001**	**0.262**	**0.042**	0.084	0.325
Outcome	**EA**		**XA**		**Control**	
Predictor	(R^2 = 0.41)		(R^2 = 0.23)		(R^2 = 0.02)	
**BDT**	*β*	*p*	*β*	*p*	*β*	*p*
Empathic Ability	**0.324**	**< .001**	**0.326**	**0.006**	0.034	0.705
Reasoning Ability	**0.406**	**< .001**	**0.275**	**0.020**	0.121	0.107
Outcome	**EA**		**XA**		**Control**	
Predictor	(R^2 = 0.07)		(R^2 = 0.04)		(R^2 = 0.06)	
**RWCA ($)**	*β*	*p*	*β*	*p*	*β*	*p*
Empathic Ability	0.100	0.352	0.184	0.158	**0.250**	**0.003**
Reasoning Ability	**-0.309**	**0.005**	-0.135	0.298	**-0.200**	**0.016**
Outcome	**EA**		**XA**		**Control**	

Predictor	(R^2 = 0.14)		(R^2 = 0.05)		(R^2 = 0.03)	
**RWCA (Time)**	*β*	*p*	*β*	*p*	*β*	*p*
Empathic Ability	0.089	0.391	0.182	0.159	0.162	0.056
Reasoning Ability	**-0.420**	**< .001**	-0.196	0.130	-0.146	0.084

Note. Bolded effects indicate statistical significance at p < .05.

Multiple Regressions Examining the Interactive Effects of Empathic and Reasoning Ability on Prosociality

Figure S5

Interactions between empathic and reasoning ability on equitable, effective, and real-world prosociality

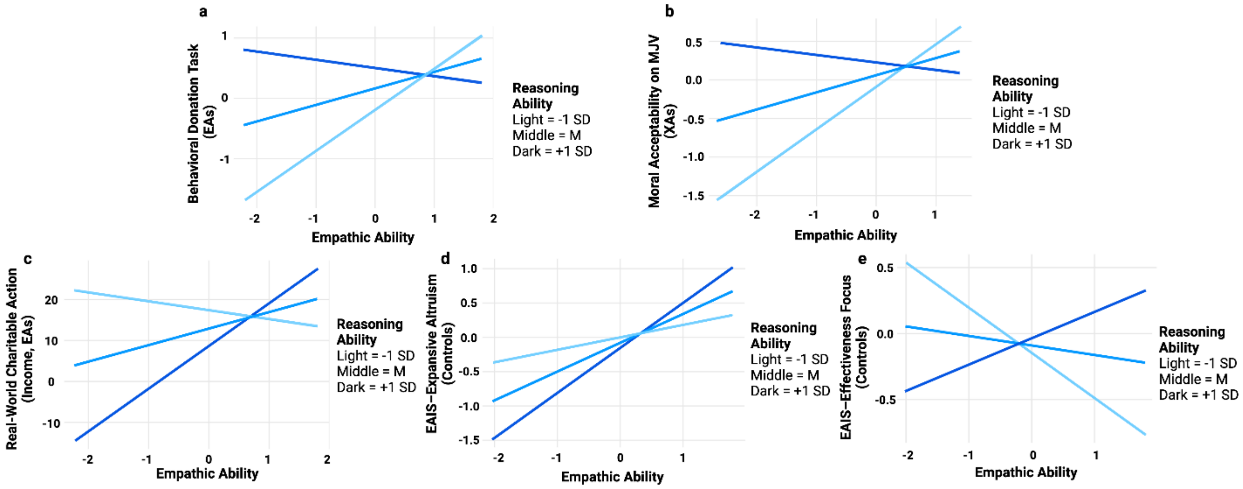

Note. Plots displaying the interaction between empathy and reasoning on prosociality among EAs on the BDT (**a**), among XAs on the MJV (**b**), among EAs on RWCA–Income (**c**), and among controls on the EAIS (**d-e**). Plots display predicted values for y across values of x.

Table S11

Results from multiple linear regression models evaluating associations between individual variation in the empathic ability factor, the reasoning ability factor, and their interaction with individual variation in equitable, effective, and real-world prosociality within Samples 1, 2, and 3

Outcome Predictor	**EA** (R^2 = 0.41)		**XA** (R^2 = 0.18)		**Control** (R^2 = 0.07)	
MJV	*β*	*p*	*β*	*p*	*β*	*p*
Empathic Ability	**0.370**	**.001**	0.168	.177	-0.046	.570
Reasoning Ability	-0.150	.158	0.156	.201	**0.283**	**< .001**
Interaction	0.004	.964	**-0.244**	**.027**	0.032	.693
Outcome Predictor	**EA** (R^2 = 0.09)		**XA** (R^2 = 0.04)		**Control** (R^2 = 0.04)	
SDT	*β*	*p*	*β*	*p*	*β*	*p*
Empathic Ability	0.198	.076	0.094	.481	**0.203**	**0.015**
Reasoning Ability	**0.277**	**.031**	0.130	.322	-0.087	0.299
Interaction	-0.076	.398	-0.066	.574	0.072	0.387
Outcome Predictor	**EA** (R^2 = 0.28)		**XA** (R^2 = 0.17)		**Control** (R^2 = 0.12)	
**EAIS-EX**	*β*	*p*	*β*	*p*	*β*	*p*
Empathic Ability	**0.385**	**<.001**	**0.315**	**.014**	**0.326**	**< .001**
Reasoning Ability	**0.217**	**.024**	0.170	.167	-0.083	.302
Interaction	0.020	.815	-0.036	.738	**.185**	**.021**
Outcome Predictor	**EA** (R^2 = 0.28)		**XA** (R^2 = 0.07)		**Control** (R^2 = 0.04)	
**EAIS-EF**	*β*	*p*	*β*	*p*	*β*	*p*
Empathic Ability	0.157	.112	0.004	.976	-0.055	.507
Reasoning Ability	**0.415**	**<.001**	0.257	.050	0.056	.501
Interaction	-0.027	.752	-0.046	.687	**0.211**	**.012**
Outcome Predictor	**EA** (R^2 = 0.50)		**XA** (R^2 = 0.26)		**Control** (R^2 = 0.02)	
**BDT**	*β*	*p*	*β*	*p*	*β*	*p*
Empathic Ability	**0.218**	**.009**	**0.292**	**.015**	0.032	.705
Reasoning Ability	**0.347**	**< .001**	**0.278**	**.019**	0.136	.107
Interaction	**-0.324**	**< .001**	-0.128	.216	-0.018	.827
Outcome Predictor	**EA** (R^2 = 0.13)		**XA** (R^2 = 0.05)		**Control** (R^2 = 0.07)	
**RWCA ($)**	*β*	*p*	*β*	*p*	*β*	*p*
Empathic Ability	0.182	.095	0.159	.235	**0.248**	**.003**
Reasoning Ability	**-0.239**	**.023**	-0.142	.279	**-0.195**	**.019**
Interaction	**0.279**	**.004**	-0.105	.369	-0.117	.153
Outcome Predictor	**EA** (R^2 = 0.13)		**XA** (R^2 = 0.09)		**Control** (R^2 = 0.04)	
**RWCA (Time)**	*β*	*p*	*β*	*p*	*β*	*p*
Empathic Ability	0.086	.429	0.149	.253	0.163	.051
Reasoning Ability	**-0.380**	**< .001**	-0.237	.067	-0.146	.081
Interaction	0.050	.600	-0.172	.131	-0.121	.147

Note. Bolded effects indicate statistical significance at $p < .05$.

Supplementary References

1. Davis, M. A Multidimensional Approach to Individual Differences in Empathy. *JSAS Catalog Sel. Doc. Psychol.* **10**, (1980).
2. Thompson, C. G., Kim, R. S., Aloe, A. M. & Becker, B. J. Extracting the Variance Inflation Factor and Other Multicollinearity Diagnostics from Typical Regression Results. *Basic and Applied Social Psychology* **39**, 81–90 (2017).
3. Bloom, P. *Against Empathy: The Case for Rational Compassion: Paul Bloom: 9780062339331: Amazon.Com: Books*. (2016).
4. Singer, P. *Famine, Affluence, and Morality*. (Oxford University Press, 2016).
5. MacAskill, W. Understanding Effective Altruism and Its Challenges. in *The Palgrave Handbook of Philosophy and Public Policy* (ed. Boonin, D.) 441–453 (Springer International Publishing, Cham, 2018). doi:10.1007/978-3-319-93907-0_34.
6. Caviola, L., Schubert, S. & Greene, J. D. The Psychology of (In)Effective Altruism. *Trends in Cognitive Sciences* (2021) doi:10.1016/j.tics.2021.03.015.
7. Marsh, A. A. & Cardinale, E. M. Psychopathy and fear: Specific impairments in judging behaviors that frighten others. *Emotion* **12**, 892–898 (2012).
8. Schumann, K., Zaki, J. & Dweck, C. S. Addressing the empathy deficit: Beliefs about the malleability of empathy predict effortful responses when empathy is challenging. *Journal of Personality and Social Psychology* **107**, 475–493 (2014).
9. Darley, J. M. & Latane, B. Bystander intervention in emergencies: Diffusion of responsibility. *Journal of Personality and Social Psychology* **8**, 377–383 (1968).
